# Fungal Diversity and Mycotoxins in Low Moisture Content Ready-To-Eat Foods in Nigeria

**DOI:** 10.3389/fmicb.2020.00615

**Published:** 2020-04-09

**Authors:** Chibundu N. Ezekiel, Oluwawapelumi A. Oyedele, Bart Kraak, Kolawole I. Ayeni, Michael Sulyok, Jos Houbraken, Rudolf Krska

**Affiliations:** ^1^Department of Microbiology, Babcock University, Ilishan Remo, Nigeria; ^2^Department of Agrobiotechnology (IFA–Tulln), Institute of Bioanalytics and Agro-Metabolomics, University of Natural Resources and Life Sciences Vienna (BOKU), Tulln, Austria; ^3^Westerdijk Fungal Biodiversity Institute, Uppsalalaan, Netherlands; ^4^Institute for Global Food Security, School of Biological Sciences, Queen’s University Belfast, Belfast, United Kingdom

**Keywords:** chemotaxonomy, citrinin, food safety, *garri*, *granola*, secondary metabolites

## Abstract

Low moisture content ready-to-eat foods vended in Nigerian markets could be pre-packaged or packaged at point of sale. These foods are widely and frequently consumed across Nigeria as quick foods. Despite their importance in the daily diets of Nigerians, a comprehensive study on the diversity of fungi, fungal metabolite production potential, and mycotoxin contamination in the foods has not yet been reported. Therefore, this study assessed the diversity of fungi in 70 samples of low moisture content ready-to-eat foods [cheese balls, *garri* (cassava-based), *granola* (a mix of cereals and nuts) and popcorn] in Nigeria by applying a polyphasic approach including morphological examination, genera/species-specific gene marker sequencing and secondary metabolite profiling of fungal cultures. Additionally, mycotoxin levels in the foods were determined by LC–MS/MS. Fungal strains (*n* = 148) were recovered only from *garri*. Molecular analysis of 107 representative isolates revealed 27 species belonging to 12 genera: *Acremonium*, *Allophoma*, *Aspergillus*, *Cladosporium*, *Fusarium*, *Microdochium*, *Penicillium*, *Sarocladium*, *Talaromyces*, and *Tolypocladium* in the Ascomycota, and *Fomitopsis* and *Trametes* in the Basidiomycota. To the best of our knowledge *Allophoma*, *Fomitopsis*, *Microdochium*, *Tolypocladium*, and *Trametes* are reported in African food for the first time. A total of 21 uncommon metabolites were found in cultures of the following species: andrastin A and sporogen AO1 in *Aspergillus flavus*; paspalin in *A. brunneoviolaceus*; lecanoic acid and rugulusovin in *A. sydowii*; sclerotin A in *P. citrinum* and *Talaromyces siamensis*; barceloneic acid, festuclavine, fumigaclavine, isochromophilons (IV, VI, and IX), ochrephilone, sclerotioramin, and sclerotiorin in *P. sclerotium*; epoxyagroclavine, infectopyron, methylorsellinic acid and trichodermamide C in *P. steckii*; moniliformin and sporogen AO1 in *P. copticola*; and aminodimethyloctadecanol in *Tolypocladium*. Twenty-four mycotoxins in addition to other 73 fungal and plant toxins were quantified in the foods. In *garri*, cheeseballs, popcorn and *granola* were 1, 6, 12, and 23 mycotoxins detected, respectively. Deoxynivalenol, fumonisins, moniliformin, aflatoxins and citrinin contaminated 37, 31, 31, 20, and 14% of all food samples, respectively. Overall, citrinin had the highest mean concentration of 1481 μg/kg in the foods, suggesting high citrinin exposures in the Nigerian populace. Fungal and mycotoxin contamination of the foods depend on pre-food and post-food processing practices.

## Introduction

Cereal and tuber crops (e.g., cassava, maize, groundnut, sorghum and wheat) contribute substantially to food security in sub-Saharan Africa (SSA) (Oguntoyinbo and Narbad, 2012; FAOSTAT, 2017; Tadesse et al., 2018). In Nigeria, these crops are produced in large quantities (FAOSTAT, 2017). However, significant portions are often lost to post-harvest catastrophe mainly due to lack of adequate handling and storage infrastructure (Bankole et al., 2006). As such, the crops are commonly processed into non-perishable forms, such as cheese balls, *garri*, *granola* and popcorn. These foods are considered as ready-to-eat (RTE), constituting additional diversity of foods for both the rural and urban populace.

**FIGURE 1:**
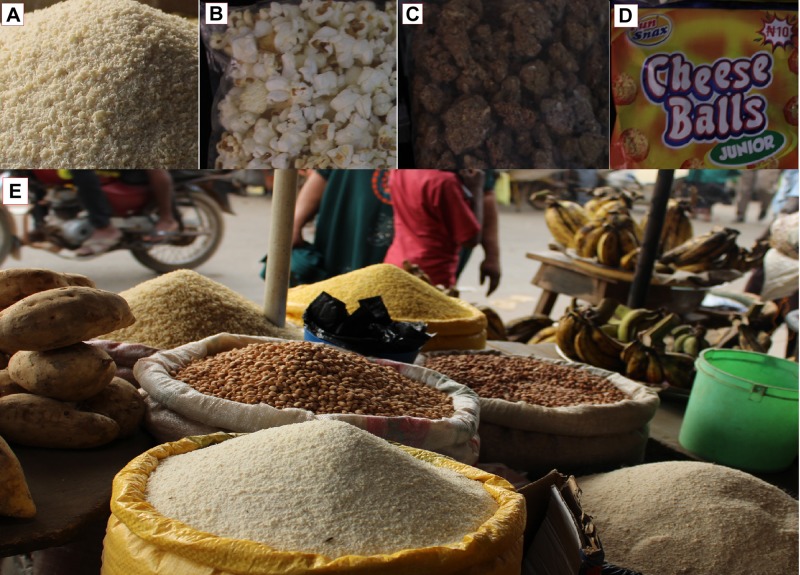
Pictorial representation of dried ready-to-eat foods **(A)** *garri*; **(B)** popcorn; **(C)**: *granola*; **(D)**: cheese balls vended in markets **(E)** market scene) in Ogun State, Nigeria.

Cheese balls are orange-colored, soft wafer-like snacks processed by local industries in Nigeria and made from corn grits and natural cheese solids. They are consumed mostly by children under age five. *Garri* is a dry, farinated, granular starchy food spontaneously produced from the fermentation and roasting (dry frying) of cassava (Okafor, 1977; Figure 1). It is a household food in many parts of West Africa, and can be consumed directly in the granulated form, or mixed with either cold or hot water. Cassava processing into *garri* mostly happens at household level and involves peeling, washing and grating cassava tubers, sack-packaging of the derived slurry, dewatering and fermentation of the packaged slurry for 2–4 days, sieving of the dewatered fermented substrate, and dry frying of sievate at approximately 100°C for 15–20 min (Okafor, 1977; Omonigho and Ikenebomeh, 2002). *Granola* is a baked, crunchy breakfast food made from mixed cereals and nuts and consumed by many households in Nigeria; Figure 1. It is produced mainly at household level but is also commercially available. The ingredients for *granola* principally include maize, groundnut, wheat, and honey or sugar. In some cases, bananas and coconut are added as supplements. Typically, *granola* processing includes mixing all ground raw ingredients in appropriate proportions and with water to form thick granules prior to baking in a pre-heated oven at about 180°C for approximately 1 h. Popcorn is a snack produced from popping dry popcorn maize mixed with honey or sugar for 5–10 min. This snack is usually consumed by all ages including children.

The safety of the aforementioned RTE foods may, however, be threatened by the presence of diverse fungi and their toxic metabolites (Makinde et al., 2020). However, the fungal and mycotoxicological safety of these foods, except for *garri* and other cassava products (Ogiehor et al., 2007; Manjula et al., 2009; Ediage et al., 2011; Thomas et al., 2012, 2017; Makun et al., 2013; Sulyok et al., 2015; Abass et al., 2017; Chilaka et al., 2018), were not previously studied in Nigeria and other African countries. This creates a gap in the fungal and mycotoxin surveillance database for commonly consumed foods in Africa. Previous studies on the fungal contamination of *garri* in Nigeria (Ogiehor et al., 2007; Thomas et al., 2012, 2017) applied either the conventional identification method or a molecular method using 18S ribosomal RNA gene sequences for identification. Both methods have several known limitations such as low precision and misleading taxonomy (Houbraken et al., 2011, 2012; Samson et al., 2014, 2019; Rico-Munoz et al., 2018). Therefore, the need to conduct a more robust fungal profiling and taxonomic studies on the foods is required. Consequently, this study aimed at a comprehensive assessment of the diversity of fungi and mycotoxin profiles in low moisture content RTE foods commonly vended in markets and widely consumed in Nigeria by applying robust polyphasic fungal taxonomic approaches and LC–MS/MS-based analysis of the foods and fungal cultures.

## Materials and Methods

### RTE Food Sampling

A total of 70 dried RTE food samples consisting of cheese balls (*n* = 10), *garri* (*n* = 23), *granola* (*n* = 18) and popcorn (*n* = 19) were randomly purchased from markets in Ogun State, Nigeria, between January and March 2018. Cheese balls, *granola* and popcorn were purchased as pre-packed foods in sealed polyethylene packs, while *garri* samples were purchased unpackaged from open vessels (basins and bags). Each sample of cheese balls and popcorn weighed approximately 200 g, whereas *garri* and *granola* samples weighed 1 kg each. Food samples were comminuted into fine powder in an electric blender (MX-AC400, Panasonic, India) and each sample was batched into three equal parts: batch A for moisture analysis, batch B for mycological analysis, and batch C for mycotoxin analysis. The batch A and B samples were stored at 4°C prior to analysis within 48 h, while batch C samples were frozen at −20°C prior to multi-mycotoxin analysis.

### Moisture Content Analysis of the Dried RTE Foods

The food samples were subjected to moisture content analysis by the oven-drying to constant weight method (AOAC, 1990). Heating temperature was set at 105°C for 1–3 h in a UNISCOPE hot air oven (SM9053, Surgifriend Medicals, England) (Commission of the European Communities, 2000) and readings were taken for triplicate samples per food sample.

### Mycological Analysis of Dried RTE Foods

#### Isolation and Enumeration of Fungi

The dilution plating technique as described by Samson et al. (1995) was applied for the isolation and enumeration of fungi present in the food samples. Each comminuted sample (10 g) was diluted in sterile distilled water (90 mL) and homogenized for 2 min. Exactly 100 μL of the homogenized mixture was surface-plated out in duplicate on Dichloran 18% Glycerol (DG18) agar. Incubation of all inoculated plates was performed at 25°C for 3 to 5 days. Thereafter, fungal colonies were counted and are reported below as colony forming units per gram (CFU/g) of analyzed food sample. All distinct colonies on the DG18 agar plates were transferred to freshly prepared plates of malt extract agar (MEA; Oxoid, United Kingdom). The purified cultures were retained at 4°C on MEA slants overlaid with sterile distilled water in 4 mL vials.

#### Polyphasic Characterization of Fungal Isolates

A polyphasic approach consisting of morphological examination, molecular typing and secondary metabolite profiling of fungal cultures was adopted for elucidating the diversity of fungi in the RTE foods. All fungal isolates obtained from the food samples were cultivated on MEA, assessed for macroscopic and microscopic characters, and compared with descriptions in appropriate keys (Frisvad and Samson, 2004; Leslie and Summerell, 2006; Pitt and Hocking, 2009; Samson et al., 2011, 2019). Isolates were clustered into phenotypic groups and representatives of each group were selected for further identification using a sequence-based approach. The representative isolates were grown on MEA for 3 to 5 d prior to DNA extraction. A part of the β-tubulin (*BenA*) and/or calmodulin (*CaM*) gene was amplified and sequenced for the isolates belonging to *Aspergillus*, *Penicillium* and *Talaromyces* as previously described (Houbraken et al., 2011, 2012; Samson et al., 2019). For other isolates, the ITS regions, a part of the translation elongation factor 1 alpha (*TEF-1*α) and/or the RNA polymerase II subunit (*RPB2*) gene were amplified and sequenced (Groenewald et al., 2005, 2013; Chen et al., 2015, 2017). Species identities were confirmed by comparing the generated sequences with sequences housed in the NCBI database and the internal curated database of the Westerdijk Fungal Biodiversity Institute (WI). All the identified isolates were deposited in the working culture collection of WI (“DTO culture collection”) and newly generated sequences are deposited in GenBank (Supplementary Table S1).

For secondary metabolite profiling, fungal cultures were cultivated on appropriate sets of media, depending on fungal genera/species, for 7 and 14 d at 25°C. The media used were Czapek yeast autolysate (CYA) agar, yeast extract sucrose (YES) agar, MEA and oatmeal (OA) agar (Yilmaz et al., 2014; Samson et al., 2019). Secondary metabolites were extracted from the cultures according to the agar plug extraction method of Filtenborg et al. (1983) with modifications from Smedsgaard (1997). Extraction of agar plugs was performed in ethylacetate/dichloromethane/methanol (3:2:1, v/v/v) containing 1% formic acid. Extracts were dried to the air prior to LC–MS/MS metabolite profiling (see section below).

### Multi-Metabolite Analysis of Agar Plug Extracts and RTE Foods

A robust dilute and shoot LC–MS/MS method described by Sulyok et al. (2020) was applied in the determination of more than 300 microbial metabolites including mycotoxins in the extracts from fungal cultures and from the food samples. The dried extracts from fungal cultures were first dissolved in 1 mL (ratio 1:1, v/v) of extraction solvent (acetonitrile/water/acetic acid 79:20:1, v/v/v) and then diluted with acetonitrile/water/acetic acid 20:79:1, v/v/v prior to injection into the LC–MS/MS instrument. For the extraction of food samples, 5 g of homogenized food was taken and mixed with 20 mL of extraction solvent in a 50 mL polypropylene tube (Sarstedt, Nümbrecht, Germany). Apparent recovery of the method was determined by spiking a multi-analyte stock solution into 0.25 g of the food samples. Spiked samples were retained overnight in order to reach equilibrium, and thereafter they were extracted with 1 mL of extraction solvent. Extraction of food samples lasted for 90 min on a GFL 3017 rotary shaker (GFL, Burgwedel, Germany). Extracts were then diluted with acetonitrile/water/acetic acid 20:79:1 (v/v/v) in a 1:1 (v/v) ratio prior to injection into the LC–MS/MS instrument (Sulyok et al., 2007).

Metabolites were screened on a QTrap 5500 LC–MS/MS System (Applied Biosystem, Foster City, CA, United States) equipped with TurboIonSpray electrospray ionisation (ESI) source and a 1290 Series HPLC System (Agilent, Waldbronn, Germany). Chromatographic separation was performed at 25°C on a Gemini^®^ C18–column, 150 × 4.6 mm i.d., 5 μm particle size, equipped with a C18 4 × 3 mm i.d. security guard cartridge (Phenomenex, Torrance, CA, United States). The chromatographic method, chromatographic and mass spectrometric parameters are as described by Sulyok et al. (2020). ESI-MS/MS was conducted in the time-scheduled multiple reaction monitoring (MRM) mode both in positive and negative polarities in two separate chromatographic runs per sample by scanning two fragmentation reactions per analyte. The MRM detection window of each analyte was set to its expected retention time ± 27 s and ± 48 s in the positive and the negative modes, respectively. The identified positive analytes were confirmed by the acquisition of two MRMs per analyte (with the exception of moniliformin (MON), which exhibited only one fragment ion). This yielded 4.0 identification points according to European Commission decision 2002/657 (European Commission [EC], 2002). Additionally, the LC retention time and the intensity ratio of the two MRM transitions were in agreement with the related values of an authentic standard within 0.1 min and 30% respectively. The accuracy of the method for food analysis was verified by participation in inter-laboratory comparison studies organized by BIPEA (Gennevilliers, France). Presently, 94% of the more than 1100 results submitted for different types of grains, nuts, dried fruits and baby food were in the satisfactory range of *z*-score between −2 and 2.

### Data Analysis

Data analysis was performed using IBM Statistical Package for SPSS 21.0 (SPSS^®^ Inc., IL, United States). Means for data on moisture content were calculated and tested for significance by One-way ANOVA (α = 0.05).

## Results and Discussion

### Moisture Content of Dried RTE Foods

The moisture levels of foods are critical for fungal contamination and mycotoxin accumulation (Magan et al., 2010). Low moisture contents (range: 2.80–10.6%; mean: 7.73 ± 2.03) were recorded in the foods analyzed in this study. The mean moisture levels of *granola* (7.47 ± 1.56; range: 5.60–10.6%), *garri* (7.90 ± 1.23; range: 2.80–9.00%) and popcorn (9.54 ± 1.13; range: 6.80–10.6%) were significantly (*p* < 0.05) higher than the mean level recorded for cheese balls (4.37 ± 1.05; range: 3.28–5.98%).

### Fungal Occurrence (Load and Incidence) in Dried RTE Foods and Their Implications

**FIGURE 2:**
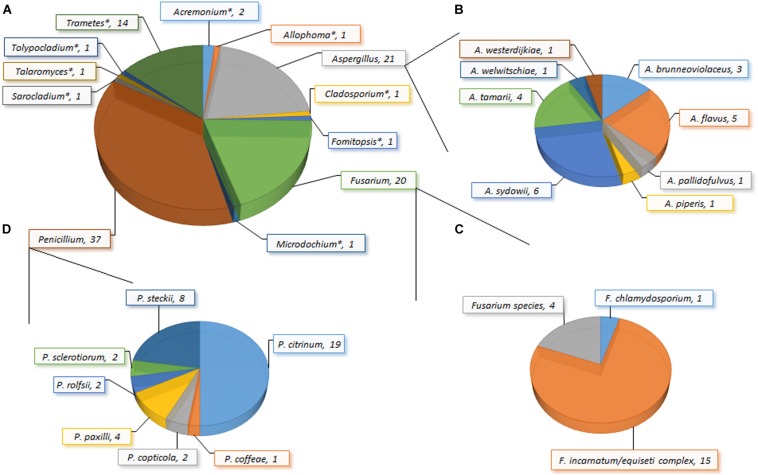
Incidence of fungi **(A)**: genera; **(B)** species within *Aspergillus*; **(C)** species within *Fusarium*; **(D)** species within *Penicillium*) in *garri* (farinated cassava) sold in markets in Ogun State, Nigeria. *Only one species of the genus was recovered: *Acremonium charticola*, *Allophoma* species, *Cladosporium tenuissimum*, *Fomitopsis meliae/durescens*, *Microdochium lycopodinum*, *Sarocladium strictum*, *Talaromyces siamensis*, *Tolypocladium* species, and *Trametes polyzona*.

Of all the food types examined in this study, only *garri* (unpackaged food from busy open markets; Figure 1) yielded fungal propagules (Figure 2). The food types (cheese balls, *granola* and popcorn) without viable fungal propagules were purchased in pre-packaged forms. This data indicate that the processing steps probably reduced the fungal levels in the food and these levels remained low due to packaging, therefore avoiding recontamination. The fungal contamination of the *garri* samples may be attributed to post-food production practices, because it is highly unlikely that the fungal propagules can survive the frying step at approximately 100°C for 15–20 min. To be precise, exposure of *garri* in the unpackaged form to the busy open markets, where human activities including constant and high human traffic and motorist influx occur for more than 10 h on a daily basis, will result in massive food contamination by diverse fungi. Thus, these open markets and the foods being sold therein (e.g., *garri*) become hot spots of transmission of pathogenic and/or toxigenic strains to new environments. Therefore, packaging after the frying step might limit fungal contamination and prevent the dissemination/dispersal of harmful strains (Guynot et al., 2003). Future research may investigate the impact of packaging (diverse materials and duration) of *garri* on fungal contamination. No visible fungal growth was observed on the investigated *garri* samples. Alternative to packaging, challenge test studies with relevant fungal strains (incl. xerophilic fungi) could be performed to study the impact of fungal contaminants on the shelf-life of *garri*.

The load of fungal propagules in the *garri* samples ranged from 200 to 2,500 (mean: 712 ± 621) CFU/g, and 148 isolates were recovered from 83% of the samples (Figure 2). The recovered fungi comprised 27 species belonging to 12 genera clustered into two taxonomic divisions (Ascomycota and Basidiomycota) based on a comprehensive taxonomic approach. *Acremonium*, *Allophoma*, *Aspergillus*, *Cladosporium*, *Fusarium*, *Microdochium*, *Penicillium*, *Sarocladium*, *Talaromyces*, and *Tolypocladium* constituted the Ascomycota, while *Fomitopsis* and *Trametes* represented the Basidiomycota. Here, we present to the best of our knowledge the first report of *Allophoma*, *Fomitopsis*, *Microdochium*, *Tolypocladium*, and *Trametes* in food in Africa (Chen et al., 2017). Several of these fungal species (e.g., *Allophoma oligotrophica* and *M. lycopodinum*) together with species belonging to *Aspergillus*, *Penicillium* and *Talaromyces* are frequent in outdoor and household/indoor air samples from different continents (Visagie et al., 2014a; Hernández-Restrepo et al., 2016; Chen et al., 2017). These airborne fungi, though commonly saprophytic, have been associated with human infections. For example, *Aspergillus sydowii* causes aspergillosis, keratomycosis and onychomycosis (Hoog, 2000), while *A. brunneoviolaceus*, *A. pallidofulvus* and the filamentous basidiomycete (*Trametes polyzona*) were previously implicated in human pulmonary (fungal lung) infection (e.g., invasive and disseminated aspergillosis) (Masih et al., 2016; Gauthier et al., 2017).

In addition, *Acremonium*, *Cladosporium* and *Sarocladium* were previously reported in cereals (maize and rice) and cocoa powder beverages in Nigeria (Makun et al., 2011; Egbuta et al., 2015). *Cladosporium* has also been reported in fruits from Nigeria, chili and *Musa* species (banana and plantains) from different countries (Bensch et al., 2018). *Acremonium* and *Sarocladium* are two phenotypically-related genera and a comprehensive phylogenetic study led to the re-allocation of some hypocrealean *Acremonium* (including the clinically important species *A. strictum*) to *Sarocladium* (Summerbell et al., 2011; Giraldo et al., 2015). The two members of the Basidiomycota recorded in the present study are wood inhabiting fungi. *Fomitopsis meliae/durescens* is a notable brown rot fungus commonly reported in the Americas, Asia and the United Kingdom (Han et al., 2016), while *Trametes polyzona* possesses ligninolytic capabilities (Lueangjaroenkit et al., 2018). These wood-related fungi may have accessed the *garri* samples during the improper storage of the food; it is a common practice for local food vendors to store their foods in improperly closed bags/containers/basins after the day’s sale together with woods collected from the farms for making fire.

*Aspergillus*, *Fusarium* and *Penicillium* were predominantly present and multiple species were recovered; all other genera were represented by single species (Figure 2). Eight *Aspergillus* species covering four sections were recovered in this study: *Circumdati* (*A. pallidofulvus*; Visagie et al., 2014c), *Flavi* (*A. flavus* and *A. tamarii*; Frisvad et al., 2019), *Nidulantes* (*A. sydowii*; Chen et al., 2016), and *Nigri* (*A. brunneoviolaceus*, *A. piperis*, *A. welwitschiae* and *A. westerdijkiae*; Samson et al., 2007; Perrone et al., 2011; Varga et al., 2011). The *Fusarium* isolates comprised two species (*F. chlamydosporum* and *F. incarnatum/equiseti* species complex; O’Donnell et al., 2013; Villani et al., 2019) and one unresolvable species reported here as *Fusarium* sp. The Penicillia were clustered into four sections: *Charlesia* (*P. coffeae*; Houbraken and Samson, 2011; Visagie et al., 2014c), *Citrina* (*P. citrinum*, *P. copticola*, *P. paxilli*, and *P. steckii*; Houbraken and Samson, 2011; Houbraken et al., 2011; Visagie et al., 2014b), *Lanata-Divaricata* (*P. rolfsii*; Houbraken and Samson, 2011), and *Sclerotiora* (*P. sclerotiorum*; Houbraken and Samson, 2011; Rivera and Seifert, 2011; Visagie et al., 2014b). Overall, *Penicillium* (incidence: 37.4%) was the predominant genus and *P. citrinum* (incidence: 18.7%) was the most frequently isolated species in the *garri* samples.

Proper characterization of fungal species is a fundamental step to understand the role of fungi in relation to food spoilage and/or possible mycotoxin production. The array of fungal species recovered from the *garri* samples in this study is more comprehensive and diverse than the previously reported spectra of species in several foods in Nigeria including *garri* (Ogiehor et al., 2007; Thomas et al., 2012, 2017), cereals (Makun et al., 2011; Egbuta et al., 2015), and nuts (Ezekiel et al., 2013, 2016; Oyedele et al., 2017) as well as in fermented foods vended in Nigerian and South African markets (Adekoya et al., 2017, 2018). The disparities in fungal species in the present study compared to the aforementioned literature may be mainly attributed to the adoption of a more robust fungal identification technique in our study. We applied specific molecular typing markers (e.g., *BenA*, *CaM*, ITS, *RPB2*, and/or *TEF-1*α) which have been shown to have high taxonomic resolving power at species (or genus) level (Groenewald et al., 2005, 2013; Houbraken et al., 2011, 2012; Chen et al., 2015, 2017; Frisvad et al., 2019; Samson et al., 2019). The sole application of ITS or 18S rRNA gene sequencing for identification of important foodborne species (e.g., *Aspergillus*, *Fusarium*, *Penicillium*, and *Talaromyces*) will lead to misidentifications and incorrect associations will be made between species on one side and produced metabolites and ecology (food) on the other.

### Secondary Metabolites in Fungal Cultures

LC-MS/MS-based chemotaxonomy, which utilizes fingerprints of secondary metabolites excreted in fungal cultures, is a valuable complementary approach during the characterization and industrial exploitation of fungi for human benefits (Frisvad and Samson, 2004; Frisvad et al., 2007, 2018; Samson et al., 2019). Strains of 20 out of the 27 fungal species identified in this study were examined for secondary metabolite production in mycological media. Cultures of strains of 17 of the examined species (excluding *Acremonium charticola*, *Penicillium coffeae* and *Sarocladium strictum*) contained diverse metabolites as shown in Table 1.

**TABLE 1:** Secondary metabolites in cultures of fungi from *garri* marketed within Ogun state, Nigeria.

Metabolites	Aspergillus brunneoviolaceus	Aspergillus flavus	Aspergillus pallidofulvus	Aspergillus piperis	Aspergillus sydow	Aspergillus tamarii	Aspergillus welwitschiae	Fusarium chlamydosporium	Fusarium incarnatum/equiseti species complex	Penicillium citrinum	Penicillium copticola	Penicillium paxilli	Penicillium rolfsii	Penicillium sclerotiorum	Penicillium steckii	Talaromyces siamensis	Tolypocladium species
16-Ketoaspergillimide	+																
3-Nitropropionic acid		+	+			+											
Aflatoxin B_1_		+															
Aflatoxin B_2_		+															
Aflatrem		+															
Aflavarin		+															
Agroclavine														+	+		
Aminodimethyloctadecanol																	+
Andrastin A		+															
Asparason A		+															
Aspergillimide	+																
Aspulvinone E				+			+										
Aspyrone			+														
Aurasperon B				+			+										
Aurasperon C				+			+										
Aurasperon G				+			+										
Aurofusarin								+									
Barceloneic acid														+			
Chanoclavin														+	+		
Chlamydospordiol								+									
Chrysogin									+								
Citreorosein	+									+		+					
Citrinin										+							
Cyclopiazonic acid		+				+											
Dechloroisochromophilon IV														+			
Deoxyfusapyron									+								
Desoxypaxillin		+										+					
Dihydrocitrinone										+							
Emodin	+				+					+		+		+			
Endocrocin	+				+					+				+			
Epoxyagroclavin															+		
Equisetin									+								
Festuclavine														+			
Fonsecin				+			+										
Fumigaclavine A														+			
Fusapyron									+								
Heptelidic acid		+															
Hydroxysydonic acid					+												
Infectopyron										+					+		
Isochromophilon IV														+			
Isochromophilon IX														+			
Isochromophilone VI														+			
Iso-Rhodoptilometrin	+									+				+			
Kojic acid		+				+											
Kotanin A		+															
Lecanoic acid					+												
Malformin A							+										
Meleagrin	+																
Methylorsellinic acid															+		
Moniliformin											+						
Neocyclocitrinol										+					+		
Nigragillin				+			+										
Notoamide derivative			+														
Notoamide E derivative			+														
NP1243										+	+				+		
NP8442										+					+		
Ochrephilone														+			
*O*-Methylsterigmatocystin		+															
Oxaline	+																
Paraherquamide E	+																
Paspalin	+	+										+					
Paspalitrem A		+															
Paspalitrem B		+															
Paxillin												+					
Penicillic acid			+										+				
Pyranonigrin				+			+										
Pyrenocin A												+					
Pyrophen							+										
Quinolactacin A										+							
Quinolactacin B										+							
Rugulusovin					+												
Scalusamid A										+							
Sclerotin A										+						+	
Sclerotioramin														+			
Sclerotiorin														+			
Secalonic acid B	+																
Secalonic acid D	+																
Secalonic acid F	+																
Sporogen AO1		+									+						
Sydonic acid					+												
Tensidol B							+										
Trichodermamide C															+		
Tryprostatin B	+		+							+			+				
Violaceol I					+												
Violaceol II					+												
Viomellein			+														
Viridicatum toxin													+				
W493B								+									
WIN-64821					+												
Xanthomegnin			+														

#### Metabolites From *Aspergillus*

Secondary metabolite production is often consistent within a species but can be highly variable within members of closely related species as well as within a section (Frisvad et al., 2007; Samson et al., 2007). Here, the metabolite patterns of three members of the section *Nigri* (*A. brunneoviolaceus*, *A. piperis*, and *A. welwitschiae*) were investigated (Table 1). *Aspergillus brunneoviolaceus* (syn. *A. fijiensis*) produced aspergillimides, emodin, meleagrin, oxaline, paraherquamide E and secalonic acids B, D, and F. Calbistrin C, pre-aurantiamin, neoxaline and okaramins (Varga et al., 2011; Vesth et al., 2018) were not detected in cultures of this species while asperparalines are not included in the target list of compounds in the LC-MS/MS method applied in the present study. *Aspergillus piperis* and *A. welwitschiae* produced distinct arrays of metabolites. *Aspergillus welwitschiae* produced malformin A, tensidol B and trace amounts of pyrophen which were not found in cultures of *A. piperis*. These metabolites from *A. welwitschiae* (syn. *A. awamori*) agree with the literature and indicate that this species shares similar metabolite profiles with *A. niger* (Samson et al., 2007; Nielsen et al., 2009; Perrone et al., 2011; Hong et al., 2013; Lamboni et al., 2016). The metabolites produced by *A. piperis* isolated from the Nigerian *garri* samples were also consistent with previous reports for naphtho-γ-pyrones (aurasperones) and pyranonigrin A (Samson et al., 2007), except for aflavinins that were not detected in cultures in the present study because the compounds are also not included in the LC-MS/MS method list of compounds. Additionally, aspulvinone E recently found in cultures of *A. niger* isolated during cocoa beans processing in Nigeria (Akinfala et al., 2020) and other notable metabolites (fonsecin and nigragillin) of the section *Nigri* (Samson et al., 2007; Akinfala et al., 2020) were also found in the *A. piperis* cultures in our present study. The chemical profile data obtained here reiterates the fact that the identification of species within section *Nigri* (black Aspergilli) is complicated, requiring great skill and the application of a polyphasic approach involving the right set of genetic markers (Samson et al., 2007; Nielsen et al., 2009; Perrone et al., 2011; Lamboni et al., 2016).

Four *A. flavus* strains were considered for metabolite profiling; three strains were aflatoxigenic while the DTO 481-G5 strain was non-aflatoxigenic but produced cyclopiazonic acid and kojic acid. The aflatoxigenic strains produced the B aflatoxins together with other known metabolites including 3-nitropropionic acid, aflatrem, aflavarins, asparason A cyclopiazonic acid, desoxypaxillin, kojic acid, kotanin A, paspalin and paspalitrems; Frisvad et al., 2019; Uka et al., 2019). We also found heptelidic acid in a culture of one aflatoxigenic *A. flavus* strain; this corroborates the findings of a previous study from our group wherein we reported this metabolite in both mycelia and culture media extracts of aflatoxigenic *A. flavus* (Kovač et al., 2020). The metabolites uncommon to *A. flavus* that were detected in this study include sporogen AO1 produced by all the four strains and andrastin A found in culture of one aflatoxigenic strain (Supplementary Figure S1). Sporogen AO1 has been documented in members of the *Aspergillus* section *Flavi* –*A. luteovirescens* and *A. oryzae*, which is the domesticated form of *A. flavus*.

Several metabolites including aspyrone, notamide, penicillic acid, viomellein and xanthomegnin were found in the *A. pallidofulvus* culture and these were consistent with literature (Visagie et al., 2014c). However, some metabolites were shared across different genera and sections. For example, penicillic acid produced by *A. pallidofulvus* cultures was also present in cultures of *Penicillium rolfsii*, whilst tryprostatin B previously reported in *A. fumigatus* (Cui et al., 1996) was found in *A. brunneoviolaceus*, *A. pallidofulvus*, *P. citrinum* and *P. rolfsii*. Additionally, emodin and endocrocin occurred in some species within *Aspergillus* sections *Nigri* and *Nidulantes* and sections *Citrina* and *Sclerotiora* of *Penicillium* (Table 1). Paspalin, a metabolite of species within the *A. flavus* clade (Frisvad et al., 2019), is reported for the first time in one strain of *A. brunneoviolaceus* (Supplementary Figure S1). Paspalin was also found in cultures of *P. paxilli*, which is in accordance with a report associating this metabolite as a stable intermediate compound in the biosynthetic pathway of paxillin in *P. paxilli* (Saikia et al., 2007). For *A. sydowii*, the sydonic acids, violaceols (I and II), WIN-64821 and several other compounds not included in the list of compounds in our LC-MS/MS method were so far documented (Chen et al., 2016; Samson et al., 2019). But here, we present the unique occurrence of lecanoic acid and rugulusovin in addition to the aforementioned compounds in the cultures of this species (Supplementary Figure S1). These compounds could represent unique fingerprints for this species since they were not yet reported in any member of the section *Nidulantes* (Chen et al., 2016; Samson et al., 2019).

#### Metabolites From *Penicillium* and *Talaromyces*

Diverse secondary metabolite profiles were detected in the cultures of the Penicillia examined in this study (Table 1). As expected, the *P. citrinum* strains biosynthesized citrinin, dihydrocitrinone and quinolactins (A and B) (Houbraken et al., 2011). These strains from *garri* appeared to be low citrinin producers (range: 3,146–3,739; mean: 3,474 μg/kg) compared to the strains from cocoa processing in Nigeria which secreted mean citrinin levels exceeding 350,000 μg/kg (Akinfala et al., 2020). Additionally, scalusamid A, which was recently reported in cultures of *P. citrinum* obtained from cocoa processing in Nigeria (Akinfala et al., 2020), were detected here together with two metabolites tentatively named NP1243 and NP8442. Few metabolites were shared among species and across sections. For instance, neocyclocitrinol was produced by *P. citrinum* and *P. steckii*, while sclerotin A (an antimicrobial compound; Curtin and Reilly, 1940) was also found for the first time in cultures of *P. citrinum* and *Talaromyces siamensis* (Supplementary Figure S1). Sclerotin A was the only metabolite in cultures of *T. siamensis* and was not previously reported in *Penicillium* section *Citrina* or in *Talaromyces* (Houbraken et al., 2011; Yilmaz et al., 2014) but is known from *Penicillium sclerotiorum* (Rivera and Seifert, 2011). *Penicillium steckii* (section *Citrina*) and *P. sclerotiorum* (section *Sclerotiora*) also shared the production of two clavine alkaloids (agroclavine and chanoclavine). However, barceloneic acid, festuclavine, fumigaclavine, isochromophilons (IV, VI and IX), ochrephilone, sclerotioramin and sclerotiorin were additional compounds in *P. sclerotiorum* cultures, and *P. steckii* produced epoxyagroclavine, infectopyron, methylorsellinic acid and trichodermamide C (Supplementary Figure S1). These aforementioned compounds were never reported yet in any of the two species (Houbraken et al., 2011; Rivera and Seifert, 2011). It is noteworthy to mention that infectopyron is usually produced by *Alternaria* species (Escrivá et al., 2017). Thus, its presence in all cultures of *P. citrinum* and *P. steckii* in the present study (Supplementary Figure S1) corroborates its recent detection in cultures of *P. citrinum* isolated from cocoa processing (Akinfala et al., 2020), and confirms production in section *Citrina*.

All the cultures of *P. paxilli* contained paxillin, desoxypaxillin and pyrenocin A as expected (Houbraken et al., 2011), while moniliformin and sporogen AO1 were detected for the first time in all the strains of *P. copticola* (Supplementary Figure S1 and Table 1). Moniliformin, a *Fusarium* mycotoxin, was first reported in *Penicillium* (*P. melanoconidium* of the section *Fasciculata*) associated with cereals in 2016 (Hallas-Møller et al., 2016). The occurrence of moniliformin in Penicillia from two sections (*Citrina* and *Fasciculata*) suggests that gene cluster responsible for the biosynthesis of this mycotoxin has a common ancestor; however, this hypothesis needs to be further investigated by whole genome sequencing of several Penicillia. Penicillic acid and viridicatum toxin were the metabolites in the culture of *P. rolfsii*.

#### Metabolites From *Fusarium* and *Tolypocladium*

The metabolites from *F. chlamydosporum* were aurofusarin, chlamydospordiol and W493B (Table 1). Contrary to expectations (Shier and Abbas, 1992; Solfrizzo et al., 1994; Munkvold, 2016), chlamydosporol was not found in the cultures of this species in our present study. For the *F. incarnatum/equiseti* species complex, chrysogin, deoxyfusapyron, fusapyron and equisetin were detected. All metabolites obtained are common to the genus *Fusarium* (Nesic et al., 2013; Broda et al., 2016; Beccari et al., 2018; Mastanjević et al., 2018). The examined *Tolypocladium* species produced only aminodimethyloctadecanol, which is reported for the first time in this species (Supplementary Figure S1). This metabolite is a sphingosine analog previously reported in only *Fusarium* species and which plays a role in apple rot (Uhlig et al., 2005; Sørensen et al., 2009).

### Fungal Secondary Metabolites and Plant Toxins in RTE Foods

**FIGURE 3:**
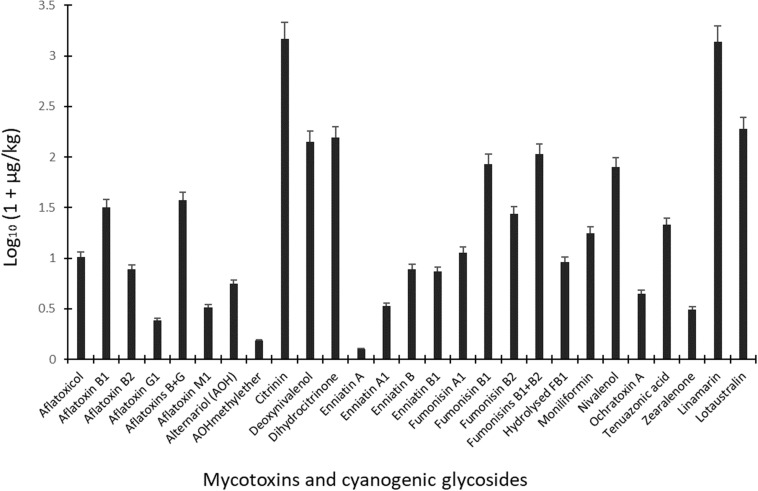
Mean levels of mycotoxins and cyanogenic glycosides in dried ready-to-eat foods vended in markets in Ogun State, Nigeria. Ochratoxin B was not included in the graph due to occurrence in only one sample.

The 70 food samples were contaminated with 95 fungal secondary metabolites and two plant toxins (cyanogenic glycosides) as quantified by the LC-MS/MS method (Table 2). Among the fungal metabolites were 24 mycotoxins commonly found in foods. The mean mycotoxin levels together with mean levels of the two cyanogenic glycosides computed for all food samples are given in Figure 3. In addition, the incidence and contamination levels of mycotoxins and cyanogenic glycosides in the food types are highlighted in Tables 3, 4, while the distribution of other fungal secondary metabolites in the food types are presented in Supplementary Table S2. At least 70% of all the food samples contained one fungal metabolite and cyanogenic glycoside. Overall, 1, 6, 12, and 23 mycotoxins were found in *garri*, cheeseballs, popcorn and *granola*. The spectrum of metabolites, including mycotoxins, found in the cereal-based and mixed grain/nut RTE foods agree with previous reports of diverse mycotoxins in processed foods of cereal, nuts or mixed cereal and nut origin (Ediage et al., 2011; Ezekiel et al., 2012, 2019; Kayode et al., 2013; Abia et al., 2017; Ojuri et al., 2018).

**TABLE 2:** LC-MS/MS method performance characteristics for 97 metabolites in dried ready-to-eat foods in Nigeria.

Metabolites		*Granola*	Popcorn	Metabolites		*Granola*	Popcorn
	LOD^a^	R^b^	R^b^		LOD^a^	R^b^	R^b^
3-Nitropropionic acid	0.80	65.6	74.7	Fumonisin B_1_	3.20	75.0	75.0
7-Hydroxypestalotin	0.40	100.7	97.8	Fumonisin B_2_	2.40	75.0	75.0
Aflatoxicol	1.00	97.7	81.8	HT-2 toxin	1.90	84.8	83.2
Aflatoxin B_1_	0.24	73.8	70.8	Hydrolyzed FB_1_	0.20	96.6	62.5
Aflatoxin B_2_	0.40	74.1	70.4	Ilicicolin A	0.02	51.8	68.7
Aflatoxin G_1_	0.32	74.8	64.4	Ilicicolin B	0.45	63.1	72.3
Aflatoxin M_1_	0.40	83.0	79.3	Ilicicolin E	0.03	92.5	86.0
Alternariol	0.40	50.1	72.8	Ilicolin C	0.20	98.4	94.1
Alternariolmethylether	0.03	83.1	77.6	Infectopyron	20.00	97.1	80.1
Ascofuranone	0.10	82.8	83.7	Iso-Rhodoptilometrin	0.03	85.4	85.2
Asperglaucide	0.08	100.0	100.0	Kojic acid	16.00	70.6	77.6
Asperphenamate	0.04	100.0	96.8	LL-Z 1272e	0.03	90.5	85.4
Aurofusarin	5.00	99.4	80.1	Linamarin	2.50	85.6	79.3
Averantin	0.04	47.4	64.9	Lotaustralin	0.10	83.1	80.8
Averufin	0.04	98.4	94.7	Macrosporin	0.13	81.1	87.8
Beauvericin	0.10	100.0	100.0	Methylsulochrin	0.04	76.2	72.8
Bikaverin	8.00	112.3	77.6	Mevinolin	1.00	102.8	81.5
Brevianamid F	0.10	61.0	61.5	Moniliformin	1.60	64.4	60.4
Chanoclavin	0.08	82.1	96.2	Monocerin	0.06	98.9	89.0
Chrysogin	0.40	69.5	66.8	Mycophenolic acid	1.10	124.8	102.2
Citreorosein	0.64	80.6	77.8	N-Benzoyl-Phenylalanine	0.15	98.5	84.9
Citrinin	0.16	72.5	49.2	Neoechinulin A	1.40	129.5	63.3
Culmorin	1.60	107.3	79.2	Nidurufin	0.16	66.0	85.9
cyclo(L-Pro-L-Tyr)	0.80	61.1	74.0	Nivalenol	0.80	93.4	89.3
cyclo(L-Pro-L-Val)	0.64	100.0	100.0	Norsolorinic acid	0.80	92.7	108.6
Cylindrol B	0.03	88.0	82.8	Ochratoxin A	0.40	107.8	90.1
Deoxynivalenol	1.00	80.0	80.8	Ochratoxin B	0.60	112.3	90.9
Dichlordiaportin	0.60	113.1	97.6	O-Methylsterigmatocystin	0.12	96.7	94.6
Dihydrocitrinone	1.00	100.0	96.0	Pestalotin	0.40	106.7	89.8
Diplodiatoxin	2.50	118.6	93.4	Phenopyrrozin	1.50	123.4	109.9
Emodin	0.06	90.1	82.8	Pinselin	0.50	101.6	85.8
Endocrocin	5.00	75.6	63.3	Purpactin A	0.70	104.1	89.8
Enniatin A	0.01	100.0	101.3	Questiomycin A	2.00	114.6	94.9
Enniatin A1	0.06	100.0	90.4	Quinolactacin A	0.08	75.4	78.6
Enniatin B	0.12	100.0	84.1	Rugulovasine A	7.60	61.0	71.1
Enniatin B1	0.08	100.0	92.7	Rugulusovin	0.7	88.9	72.6
Epiequisetin	0.24	278.0	172.9	Secalonic acid D	4.00	114.0	85.4
Equisetin	0.24	193.3	151.9	Skyrin	0.08	38.3	56.1
Ergocornine	0.90	80.8	57.3	Sterigmatocystin	0.10	93.3	84.1
Ergocristine	0.40	124.9	79.3	T-2 toxin	0.80	100.8	87.2
Ergocristinine	0.25	94.1	74.1	Tentoxin	0.10	107.7	101.6
Ergometrine	1.10	100.0	104.7	Tenuazonic acid	10.00	240.1	189.1
Ergometrinine	0.03	84.1	85.8	Terphenyllin	2.00	90.8	70.5
Ergotamine	0.90	71.9	73.0	Territrem B	0.60	113.4	88.8
Ergotaminine	0.40	61.7	55.3	Tryptophol	4.00	76.9	62.1
Fallacinol	0.10	73.4	76.5	Versicolorin A	0.24	78.5	85.1
Fellutanine A	1.00	100.0	94.3	Versicolorin C	0.24	68.7	89.5
Flavoglaucin	0.24	100.0	100.0	Zearalenone	0.20	84.1	77.9
Fumonisin A_1_	3.20	75.0	75.0				

*^*a*^Limit of detection: expressed as μg/kg sample; S/N = 3:1. ^*b*^Recovery from spiking food samples (*n* = 5). LC-MS/MS performance parameters for *garri* are as given in Abass et al. (2017).*

**TABLE 3:** Occurrence of 26 mycotoxins and cyanogenic glucosides in dried cereal-based ready-to-eat foods from Ogun state, Nigeria.

Mycotoxins	*Granola* (*n* = 18)						Popcorn (*n* = 19)					
	*N* (%)^a^	Min	Max	Median	Mean	SD	*N* (%)^a^	Min	Max	Median	Mean	SD
Aflatoxicol	2 (11.1)	6.62	11.7	9.17	9.17	3.60	0 (0)	<LOD	<LOD	<LOD	<LOD	<LOD
Aflatoxin B_1_	14 (77.8)	0.48	104	16.4	30.9	36.7	0 (0)	<LOD	<LOD	<LOD	<LOD	<LOD
Aflatoxin B_2_	11 (61.1)	0.54	20.8	3.30	6.72	6.92	0 (0)	<LOD	<LOD	<LOD	<LOD	<LOD
Aflatoxin G_1_	5 (27.8)	0.40	4.32	0.62	1.42	1.65	0 (0)	<LOD	<LOD	<LOD	<LOD	<LOD
Aflatoxin M_1_	7 (38.9)	1.17	2.99	2.50	2.27	0.61	0 (0)	<LOD	<LOD	<LOD	<LOD	<LOD
Alternariol (AOH)	2 (11.1)	1.08	8.03	4.55	4.55	4.91	0 (0)	<LOD	<LOD	<LOD	<LOD	<LOD
AOHmethylether	16 (88.9)	0.24	1.85	0.36	0.48	0.38	5 (26.3)	0.49	1.01	0.69	0.74	0.20
Beauvericin	0 (0)	<LOD	<LOD	<LOD	<LOD	<LOD	1 (5.3)	7.93	7.93	7.93	7.93	–
Citrinin	10 (55.6)	7.25	4415	792	1481	1764	0 (0)	<LOD	<LOD	<LOD	<LOD	<LOD
Deoxynivalenol	18 (100)	72.1	325	149	158	73.5	7 (36.8)	21.0	286	55.6	112	114
Dihydrocitrinone	7 (38.9)	4.48	293	179	155	116	0 (0)	<LOD	<LOD	<LOD	<LOD	<LOD
Enniatin A	18 (100)	0.13	0.82	0.23	0.26	0.15	0 (0)	<LOD	<LOD	<LOD	<LOD	<LOD
Enniatin A1	18 (100)	1.28	7.84	2.05	2.37	1.44	0 (0)	<LOD	<LOD	<LOD	<LOD	<LOD
Enniatin B	18 (100)	2.61	31.5	4.10	7.55	7.56	2 (10.5)	0.14	0.17	0.15	0.15	0.02
Enniatin B1	18 (100)	3.22	27.2	5.22	7.11	5.80	2 (10.5)	0.22	0.33	0.27	0.27	0.08
Fumonisin A_1_	4 (22.2)	5.04	11.3	6.28	7.23	2.86	3 (15.8)	8.43	22.4	13.2	14.7	7.10
Fumonisin B_1_	14 (77.8)	15.1	128	78.1	74.0	31.2	7 (36.8)	32.5	239	120	114	82.1
Fumonisin B_2_	13 (72.2)	8.96	39.3	25.8	25.2	8.51	5 (26.3)	11.2	47.1	36.0	29.6	15.3
Hydrolyzed FB_1_	4 (22.2)	4.33	13.1	9.16	8.94	3.60	1 (5.3)	4.95	4.95	4.95	4.95	–
Lotaustralin	4 (22.2)	16.8	24.9	18.5	19.7	3.58	0 (0)	<LOD	<LOD	<LOD	<LOD	<LOD
Moniliformin	17 (94.4)	4.02	29.6	10.5	12.1	6.24	4 (21.1)	6.45	79.0	22.1	32.4	32.4
Nivalenol	0 (0)	<LOD	<LOD	<LOD	<LOD	<LOD	4 (21.1)	23.0	130	80.1	78.2	53.8
Ochratoxin A	5 (27.8)	0.66	8.28	3.38	3.47	3.00	0 (0)	<LOD	<LOD	<LOD	<LOD	<LOD
Ochratoxin B	1 (5.6)	3.57	3.57	3.57	3.57	-	0 (0)	<LOD	<LOD	<LOD	<LOD	<LOD
Tenuazonic acid	13 (72.2)	4.93	31.2	20.8	19.4	7.95	0 (0)	<LOD	<LOD	<LOD	<LOD	<LOD
Zearalenone	11 (61.1)	0.81	5.99	1.33	1.73	1.47	1 (5.3)	6.40	6.40	6.40	6.40	–

*^*a*^Number (percentage) of contaminated samples.*

**TABLE 4:** Mycotoxin and cyanogenic glucoside levels in *garri* (farinated cassava; *n* = 23) samples from Ogun state, Nigeria.

Toxins	*N* (%)^a^	Range	Median	Mean	Standard deviation
Alternariolmethylether	4 (17.4)	0.2–0.7	0.4	0.4	0.22
Linamarin	22 (95.7)	87.8–8960	445	1388	2312
Lotaustralin	22 (95.7)	17.8–1630	73.5	220	420

*^*a*^Number (percentage) of contaminated samples.*

Due to the occurrence of mycotoxins [deoxynivalenol (20.4 μg/kg), fumonisin B_1_ (25.9 μg/kg), HT-2 toxin (21.6 μg/kg), moniliformin (32.6 μg/kg), T-2 toxin (20.3 μg/kg) and tenuazonic acid (32.5 μg/kg)] in only one sample of cheese balls, the data for this food is not presented in the tables. However, in view of the cocktail of toxic fungal metabolites found in cheese balls and the fact that this food is consumed mostly by pre-school and early school-aged children (under age 5), more surveillance studies are required to understand the extent of contamination in this food and possible co-exposure patterns (Ojuri et al., 2019). Aflatoxins contaminated 20% of all food samples at mean total B and G aflatoxin levels of 36.6 μg/kg (range: 0.48–118 μg/kg). Citrinin and its hydroxylated metabolite (dihydrocitrinone) were quantified in 14 and 10% of the food samples and at high mean concentrations of 1,481 μg/kg (range: 7.25–4,415 μg/kg) and 155 μg/kg (range: 4.48–293 μg/kg), respectively (Figure 3). Amongst all mycotoxins quantified in the food samples, citrinin had the highest mean level. Citrinin is a nephrotoxin presently classed as group 3 compound (International Agency for Research on Cancer [IARC], 1993) because of insufficient evidence for carcinogenicity. In addition, legal requirement or regulation is unavailable for this food in the instance of surveillance activities; thus, limited dietary exposure data abound (Ali and Degen, 2019). However, the European Food Safety Authority (EFSA) has set a 0.2 μg/kg bw/day “level of no concern for nephrotoxicity” in humans based on recent data available and given uncertainties (European Food Safety Authority [EFSA], 2012; Ali and Degen, 2019). The mean citrinin and dihydrocitrinone concentrations in the commonly consumed foods analyzed in the present study are remarkably high, suggesting high citrinin exposures in the Nigerian populace. This view is supported by recent biomonitoring data in Nigeria where citrinin and dihydrocitrinone were found in human urines (Šarkanj et al., 2018) and estimations presented high citrinin intake and exposure exceeding the set preliminary tolerable daily intake value by EFSA (Ali and Degen, 2019). More surveillance and human biomonitoring studies are required to understand the extent of citrinin contamination and dietary exposure as well as the patterns of its co-exposures with other chemical contaminants in Nigeria.

With respect to mycotoxins in individual food types, aflatoxins, citrinin, dihydrocitrinone, enniatins A and A1, and ochratoxins A and B were mycotoxins found only in *granola* in addition to other mycotoxins also found in the other foods (Table 3). Additionally, fumonisins (mean FB_1_ + FB_2_: 106 μg/kg; range: 15.1–286 μg/kg) were quantified in 31% of all 70 food samples (Figure 3), occurring only in 1, 14, and 7 samples of cheese balls, *granola* (mean FB_1_ + FB_2_: 97.5 μg/kg) and popcorn (mean FB_1_ + FB_2_: 135 μg/kg), respectively (Table 3). Deoxynivalenol (mean: 140 μg/kg; range: 20.4–325 μg/kg) and moniliformin (mean: 16.7 μg/kg; range: 4.02–78.9 μg/kg) also contaminated at least 30% of all food samples and occurred in all foods except *garri*. The occurrence of mycotoxins in cheese balls, *granola* and popcorn were not previously reported in Africa. However, the raw ingredients used in making these foods have been reported to contain mycotoxins similar to those reported in the finished foods (Warth et al., 2012; Abia et al., 2013; Adetunji et al., 2014; Matumba et al., 2015; Egbontan et al., 2017; Oyedele et al., 2017). Nonetheless, dos Santos et al. (2015) reported aflatoxins in 60% of 60 samples of *granola* in Teresina (Brazil), while Moreira et al. (2016) did not find any aflatoxins in three *granola* samples in northeastern regions of Brazil. In addition, aflatoxins were either not detected or were found together with ochratoxin A in 7 to 33% of popcorn samples in southern Pennsylvania (United States) or in popcorn imported into Spain from the United States, respectively (Alborch et al., 2012; Gourama, 2015). Thus, these commercial foods as well as other RTE foods or snacks highly consumed by children in Nigeria require routine monitoring. In Nigeria, only aflatoxins are regulated in foods at present and at a limit of 4 μg/kg. Even though aflatoxin was not detected in the other RTE food samples examined in this study, this toxin was detected in *granola* which serves as breakfast cereal in many homes. The detection of several mycotoxins regulated in the European Union, coupled with the documented toxicological effects of these mycotoxins in addition to their potential combinatory effects with the non-regulated toxins (Ostry, 2009; Klarić et al., 2013; Lee et al., 2015; Rychlik et al., 2016; Vejdovszky et al., 2016, 2017a,b; European Food Safety Authority [EFSA], 2018), and the routine high consumption of mixtures of all the examined foods together with other high risk foods in households, especially among children, necessitate a need for the urgent revision of regulations on industrially-processed foods consumed by children in Nigeria. The revision should prioritize the inclusion of other mycotoxins beyond aflatoxins in the regulations as well as setting of reasonable limits.

Linamarin and lotaustralin (two cyanogenic glycosides) were the major compounds in 96% of the 23 *garri* samples (Table 4), at mean concentrations of 1388 and 220 μg/kg, respectively. Concentrations of the two cyanogenic glycosides in the *garri* samples were obviously below the regulations for the toxic cyanides in food (Food and Agricultural Organization of the United Nations [FAO] and World Health Organization [WHO], 1992). Similar compliant levels of cyanogenic glycosides were also reported in cassava products including *garri* in Nigeria (Abass et al., 2017) but negates the high levels documented in processed cassava samples from Tanzania (Sulyok et al., 2015), obviously due to differences in cyanide contents of cassava varieties and/or differences in adopted processing methods in the two countries.

Alternariolmethylether was the only mycotoxin found in 4 *garri* samples, albeit at low concentrations (mean: 0.4 μg/kg; range: 0.2–0.7 μg/kg; Table 4) that may not obviously constitute a threat to the safety of the food. The non-detection of major mycotoxins in the *garri* samples we analyzed negates previous reports on the presence of aflatoxins, deoxynivalenol, fumonisins, moniliformin, T-2 toxin and zearalenone in cassava products including *garri* from various African countries including Nigeria (Essono et al., 2009; Sulyok et al., 2015; Abass et al., 2017; Chilaka et al., 2018). Since mycotoxins are uncommon in fresh tubers (e.g., cassava and yam) and stored tuber products could contain mycotoxins as reported in the literature, the disparity in our results from those that reported mycotoxins in cassava products may be attributed to two major factors. These include: possible low moisture content (range: 2.80–9.00%) resulting from adequate dry frying of the *garri* samples and short storage time (less than 2 weeks) of the samples analyzed in the present study compared to the other reports wherein these details were not provided. However, when the levels of the toxins in *garri* from all the studies are compared, *garri* may be regarded as a safe food in terms of mycotoxin contamination due to the low concentrations (within safe limits for processed foods) in the samples. However, this high carbohydrate/energy food may constitute a bigger health risk in the present unpackaged form in which it is vended. Unpackaged foods, as seen in the case of *garri*, could harbor fungal propagules from the air. Thus, these foods may serve as vehicle for the transmission of the propagules into environments with individuals that are susceptible for fungal infections (Hoog, 2000; Visagie et al., 2014c; Masih et al., 2016; Gauthier et al., 2017). Additionally, unpackaged *garri* contaminated with fungal propagules and stored for days in consumer household stores could serve as vehicle for fungal spore dispersal into other foods co-stored within the households and even to household/indoor air. Furthermore, in accordance with previous reports and findings from our present study, it is obvious that further mycotoxin research of cassava products including the exported ones (e.g., *garri*, cassava balls and chips to Europe) is required.

## Conclusion

Low moisture ready-to-eat foods have been shown in this study to harbor diverse fungal species and genera, including potential industrial workhorses, mushrooms, human pathogens and notable mycotoxin producers. We have also presented a rich diversity in the secondary metabolite profiles from several fungal species. Several of the presented metabolites were not previously reported in the screened species and several of them may serve as markers for species chemotaxonomy. In addition, the mycotoxin contamination profiles and levels in the foods were examined and found to be of potential public health concern, especially the mixed cereal food (*granola*). Furthermore, this study has revealed that pre-food production practices (e.g., crop cultivation, harvesting, cleaning, drying and storage) were responsible for mycotoxin levels in the RTE foods, whilst post-production food handling (e.g., food packaging *vs* non-packaging) influenced the fungal diversity in the RTE foods, though none of the foods were visibly molded. Consequently, a set of simple interventions to curb fungal and mycotoxin exposure through these foods is hereby proposed: (a) ensure proper grain cultivation, timely harvesting, safe grain cleaning, drying to safe moisture levels, and storage of grains under safe/non-damp conditions; (b) careful hand sorting of grains for production of *granola* or other foods to remove physically damaged, insect infested, discolored and moldy grains; and (c) application of simple polyethylene pre-packaging to all low moisture foods retailed in local markets in Nigeria.

## Data Availability Statement

The datasets generated for this study can be found in the GenBank, Please see Supplementary Table S1 for sequence details.

## Author Contributions

CE conceived the study. CE, JH, KA, MS, OO, and RK designed the study. BK, CE, JH, MS, and OO performed the experiments in Austria, Nigeria, and Netherlands. CE, JH, and MS analyzed the data. JH and RK supervised the overall study. CE, KA, and OO drafted the manuscript. All authors reviewed and approved the manuscript.

## Conflict of Interest

The authors declare that the research was conducted in the absence of any commercial or financial relationships that could be construed as a potential conflict of interest.
