# Supplementary material for: Fungal Diversity and Mycotoxins in Low Moisture Content Ready-To-Eat Foods in Nigeria
Source: Front Microbiol. 2020 Apr 9;11:615. doi: 10.3389/fmicb.2020.00615 (PMC7161469; doi:10.3389/fmicb.2020.00615)
Supplement: Supplementary file 1 [file Data_Sheet_1.docx]

**Fungal diversity and mycotoxins in low moisture content ready-to-eat foods in Nigeria**

**Running title: Fungi and mycotoxins in ready-to-eat foods**

Chibundu N. Ezekiel^1,2,*^, Oluwawapelumi A. Oyedele^1^, Bart Kraak^3^, Kolawole I. Ayeni^1^, Michael Sulyok^2^, Jos Houbraken^3^ and Rudolf Krska^2,4^

*^1^Department of Microbiology, Babcock University, Ilishan Remo, Ogun State, Nigeria. ^2^Institute of Bioanalytics and Agro–Metabolomics, Department of Agrobiotechnology (IFA–Tulln), University of Natural Resources and Life Sciences Vienna (BOKU), Konrad Lorenzstr. 20, A–3430 Tulln, Austria. ^3^Westerdijk Fungal Biodiversity Institute, Uppsalalaan 8, 3584 CT Utrecht, The Netherlands. ^4^Institute for Global Food Security, School of Biological Sciences, Queen’s University Belfast, University Road, Belfast, BT7 1NN, Northern Ireland, United Kingdom.*

*Corresponding author e–mail: [chaugez@gmail.com](mailto:chaugez@gmail.com) (C.N. Ezekiel)

**SUPPLEMENTARY TABLES**

**Supplementary Table 1.** Origin and sequence details of fungal isolates tested for secondary metabolite production.

| DTO^a^ number | Identity | Substrate | Location | GenBank accession numbers^b^ | | | | |
| --- | --- | --- | --- | --- | --- | --- | --- | --- |
|  |  |  |  | *BenA* | *CaM* | ITS | *TEF* | *RPB2* |
| DTO 417-E4 | *Aspergillus brunneoviolaceus* | *Garri* | Shagamu market, Ogun state | MN882768 | MN882796 | - | - | - |
| DTO 417-E7 | *Aspergillus brunneoviolaceus* | *Garri* | Shagamu market, Ogun state | MN882769 | MN882797 | - | - | - |
| DTO 417-E9 | *Aspergillus sydowii* | *Garri* | Shagamu market, Ogun state | MN882771 | MN882799 | - | - | - |
| DTO 417-F1 | *Aspergillus brunneoviolaceus* | *Garri* | Shagamu market, Ogun state | MN882770 | MN882798 | - | - | - |
| DTO 417-F2 | *Fusarium incarnatum/equiseti* complex | *Garri* | Shagamu market, Ogun state | - | - | MN882828 | MN882764 | MN882826 |
| DTO 417-F3 | *Aspergillus sydowii* | *Garri* | Shagamu market, Ogun state | MN882772 | MN882800 | - | - | - |
| DTO 417-F5 | *Aspergillus welwitschiae* | *Garri* | Shagamu market, Ogun state | MN882773 | MN882801 | - | - | - |
| DTO 417-F7 | *Aspergillus flavus* | *Garri* | Shagamu market, Ogun state | MN882774 | MN882802 | - | - | - |
| DTO 417-F8 | *Aspergillus flavus* | *Garri* | Shagamu market, Ogun state | MN882775 | MN882803 | - | - | - |
| DTO 417-H6 | *Penicllium steckii* | *Garri* | Shagamu market, Ogun state | MN882776 | MN882804 | - | - | - |
| DTO 417-H8 | *Aspergillus tamarii* | *Garri* | Shagamu market, Ogun state | MN882777 | MN882805 | - | - | - |
| DTO 417-H9 | *Talaromyces siamensis* | *Garri* | Ilishan market, Ogun state | MN882778 | MN882806 | - | - | - |
| DTO 417-I1 | *Penicillium rolfsii* | *Garri* | Ilishan market, Ogun state | MN882779 | MN882807 | - | - | - |
| DTO 417-I5 | *Penicllium steckii* | *Garri* | Ilishan market, Ogun state | MN882780 | MN882808 | - | - | - |
| DTO 417-I7 | *Penicillium sclerotiorum* | *Garri* | Ilishan market, Ogun state | MN882781 | MN882809 | - | - | - |
| DTO 417-I8 | *Fusarium incarnatum/equiseti* complex | *Garri* | Ilishan market, Ogun state | - | - | MN882829 | MN882765 | MN882823 |
| DTO 418-A2 | *Penicillium paxilli* | *Garri* | Oke Ata market, Abeokuta, Ogun state | MN882782 | MN882810 | - | - | - |
| DTO 418-B1 | *Penicillium coffeae* | *Garri* | Shagamu market, Ogun state | MN882783 | MN882811 | - | - | - |
| DTO 418-B2 | *Acremonium charticola* | *Garri* | Ilishan market, Ogun state | - | - | MN882830 | - | MN882824 |
| DTO 418-B4 | *Penicillium paxilli* | *Garri* | Shagamu market, Ogun state | MN882784 | MN882812 | - | - | - |
| DTO 418-B5 | *Aspergillus tamarii* | *Garri* | Ilishan market, Ogun state | MN882785 | MN882813 | - | - | - |
| DTO 418-B7 | *Aspergillus pallidofulvus* | *Garri* | Ijebu Ode market, Ogun state | MN882786 | MN882814 | - | - | - |
| DTO 418-B8 | *Aspergillus flavus* | *Garri* | Ijebu Ode market, Ogun state | MN882787 | MN882815 | - | - | - |
| DTO 418-B9 | *Penicillium citrinum* | *Garri* | Ijebu Ode market, Ogun state | MN882788 | MN882816 | - | - | - |
| DTO 418-C1 | *Fusarium chlamydosporum* | *Garri* | Ijebu Ode market, Ogun state | - | - | MN882831 | MN882766 | MN882825 |

**Supplementary Table 1.** Continued.

| DTO^a^ number | Identity | Substrate | Location | GenBank accession numbers^b^ | | | | |
| --- | --- | --- | --- | --- | --- | --- | --- | --- |
|  |  |  |  | *BenA* | *CaM* | ITS | *TEF* | *RPB2* |
| DTO 418-E3 | *Penicillium citrinum* | *Garri* | Shagamu market, Ogun state | MN882789 | MN882817 | - | - | - |
| DTO 418-E4 | *Aspergillus piperis* | *Garri* | Shagamu market, Ogun state | MN882790 | MN882818 | - | - | - |
| DTO 418-E8 | *Tolypocladium* sp. | *Garri* | Ilishan market, Ogun state | - | - | MN882832 | - |  |
| DTO 418-F1 | *Penicillium citrinum* | *Garri* | Ilishan market, Ogun state | MN882791 | MN882819 | - | - | - |
| DTO 418-F2 | *Penicillium copticola* | *Garri* | Ilishan market, Ogun state | MN882792 | MN882820 | - | - | - |
| DTO 418-F3 | *Penicillium copticola* | *Garri* | Ijebu Ode market, Ogun state | MN882793 | MN882821 | - | - | - |
| DTO 418-G5 | *Aspergillus flavus* | *Garri* | Shagamu market, Ogun state | MN882794 | MN882822 | - | - | - |

^a^Working culture collection of Westerdijk Fungal Biodiversity Institute (“DTO culture collection”).

^b^GenBank Accession numbers are based on sequences from amplified β-tubulin (*BenA*), calmodulin (*CaM*), internal transcribed spacer (ITS), translation elongation factor 1 alpha (*TEF-1α*), RNA polymerase II subunit (*RPB2*) genes.

**Supplementary Table 2.** Distribution of fungal metabolites in dried ready-to-eat foods from Ogun state, Nigeria.

| Metabolites | Garri (*n*=23) | | | | |  | *Granola* (*n*=18) | | | | |  | Popcorn (*n*=19) | | | | |
| --- | --- | --- | --- | --- | --- | --- | --- | --- | --- | --- | --- | --- | --- | --- | --- | --- | --- |
|  | %P^a^ | Min | Max | Mean | Median |  | %P^a^ | Min | Max | Mean | Median |  | %P^a^ | Min | Max | Mean | Median |
| 3-NPA^c^ | 0.0 | <LOD | <LOD | <LOD | <LOD |  | 55.6 | 4.65 | 135 | 37.0 | 9.33 |  | 5.3 | 11.1 | 11.1 | 11.1 | 11.1 |
| 7-HydroxyPEST^c^ | 0.0 | <LOD | <LOD | <LOD | <LOD |  | 61.1 | 1.59 | 8.00 | 4.08 | 2.92 |  | 5.3 | 6.60 | 6.60 | 6.60 | 6.60 |
| Asperglaucide | 73.9 | 0.91 | 3.86 | 2.02 | 1.63 |  | 100 | 1.15 | 581 | 110 | 36.8 |  | 100 | 3.48 | 914 | 286 | 211 |
| Asperphenamate | 34.8 | 0.68 | 4.68 | 1.76 | 1.14 |  | 100 | 1.73 | 112 | 18.9 | 4.08 |  | 63.2 | 1.15 | 32.7 | 10.6 | 4.78 |
| Aurofusarin | 0.0 | <LOD | <LOD | <LOD | <LOD |  | 5.6 | 11.2 | 11.2 | 11.2 | 11.2 |  | 21.1 | 24.2 | 62.4 | 44.5 | 45.8 |
| Averantin | 0.0 | <LOD | <LOD | <LOD | <LOD |  | 27.8 | 0.47 | 0.84 | 0.68 | 0.70 |  | 0.0 | <LOD | <LOD | <LOD | <LOD |
| Averufin | 4.3 | 0.17 | 0.17 | 0.17 | 0.17 |  | 77.8 | 0.12 | 6.24 | 1.33 | 0.85 |  | 0.0 | <LOD | <LOD | <LOD | <LOD |
| Bikaverin | 0.0 | <LOD | <LOD | <LOD | <LOD |  | 44.4 | 8.96 | 206 | 46.9 | 19.7 |  | 36.8 | 9.71 | 55.8 | 25.4 | 24.7 |
| Brevianamid F | 100 | 30.2 | 304 | 130 | 108 |  | 100 | 14.8 | 44.3 | 26.3 | 25.4 |  | 100 | 9.19 | 39.0 | 23.7 | 22.1 |
| Chanoclavin | 0.0 | <LOD | <LOD | <LOD | <LOD |  | 22.2 | 0.22 | 0.91 | 0.60 | 0.63 |  | 5.3 | 0.55 | 0.55 | 0.55 | 0.55 |
| Chrysogin | 0.0 | <LOD | <LOD | <LOD | <LOD |  | 88.9 | 2.12 | 6.35 | 4.03 | 3.87 |  | 5.3 | 12.1 | 12.1 | 12.1 | 12.1 |
| Citreorosein | 0.0 | <LOD | <LOD | <LOD | <LOD |  | 50.0 | 1.99 | 12.4 | 6.53 | 6.54 |  | 0.0 | <LOD | <LOD | <LOD | <LOD |
| Culmorin | 0.0 | <LOD | <LOD | <LOD | <LOD |  | 100 | 40.0 | 192 | 107 | 101 |  | 26.3 | 128 | 167 | 141 | 135 |
| cyclo(L-Pro-L-Tyr) | 100 | 57.7 | 707 | 293 | 250 |  | 100 | 53.2 | 261 | 124 | 116 |  | 100 | 76.3 | 204 | 137 | 132 |
| cyclo(L-Pro-L-Val) | 100 | 87.8 | 2882 | 1416 | 1465 |  | 100 | 58.6 | 685 | 211 | 164 |  | 100 | 80.6 | 285 | 177 | 176 |
| Cylindrol B | 0.0 | <LOD | <LOD | <LOD | <LOD |  | 5.6 | 0.14 | 0.14 | 0.14 | 0.14 |  | 0.0 | <LOD | <LOD | <LOD | <LOD |
| Dichlordiaportin | 0.0 | <LOD | <LOD | <LOD | <LOD |  | 11.1 | 9.09 | 10.4 | 9.76 | 9.76 |  | 15.8 | 10.2 | 55.1 | 29.6 | 23.6 |
| Diplodiatoxin | 0.0 | <LOD | <LOD | <LOD | <LOD |  | 0.0 | <LOD | <LOD | <LOD | <LOD |  | 15.8 | 41.1 | 227 | 118 | 84.1 |
| Emodin | 30.4 | 0.48 | 1.26 | 0.75 | 0.67 |  | 100 | 1.07 | 10.9 | 3.13 | 2.16 |  | 31.6 | 1.09 | 4.38 | 1.85 | 1.33 |
| Endocrocin | 0.0 | <LOD | <LOD | <LOD | <LOD |  | 61.1 | 21.5 | 67.2 | 36.2 | 31.4 |  | 5.3 | 76.9 | 76.9 | 76.9 | 76.9 |
| Epiequisetin | 0.0 | <LOD | <LOD | <LOD | <LOD |  | 100 | 0.47 | 2.99 | 1.31 | 1.16 |  | 26.3 | 1.04 | 4.86 | 2.28 | 1.73 |
| Equisetin | 0.0 | <LOD | <LOD | <LOD | <LOD |  | 100 | 0.64 | 5.28 | 2.16 | 1.73 |  | 31.6 | 0.50 | 11.4 | 4.85 | 4.04 |
| Ergocornine | 0.0 | <LOD | <LOD | <LOD | <LOD |  | 11.1 | 1.04 | 2.14 | 1.59 | 1.59 |  | 0.0 | <LOD | <LOD | <LOD | <LOD |
| Ergocristine | 0.0 | <LOD | <LOD | <LOD | <LOD |  | 77.8 | 1.41 | 5.16 | 2.75 | 2.65 |  | 0.0 | <LOD | <LOD | <LOD | <LOD |
| Ergocristinine | 0.0 | <LOD | <LOD | <LOD | <LOD |  | 83.3 | 0.67 | 3.21 | 1.57 | 1.71 |  | 0.0 | <LOD | <LOD | <LOD | <LOD |
| Ergometrine | 0.0 | <LOD | <LOD | <LOD | <LOD |  | 88.9 | 2.18 | 15.1 | 8.20 | 8.04 |  | 0.0 | <LOD | <LOD | <LOD | <LOD |
| Ergometrinine | 0.0 | <LOD | <LOD | <LOD | <LOD |  | 88.9 | 0.05 | 0.24 | 0.13 | 0.12 |  | 0.0 | <LOD | <LOD | <LOD | <LOD |

**Supplementary Table 2.** Continued.

| Metabolites | Garri (*n*=23) | | | | |  | *Granola* (*n*=18) | | | | |  | Popcorn (*n*=19) | | | | |
| --- | --- | --- | --- | --- | --- | --- | --- | --- | --- | --- | --- | --- | --- | --- | --- | --- | --- |
|  | %P^a^ | Min | Max | Mean | Median |  | %P^a^ | Min | Max | Mean | Median |  | %P^a^ | Min | Max | Mean | Median |
| Ergotamine | 0.0 | <LOD | <LOD | <LOD | <LOD |  | 66.7 | 1.62 | 5.02 | 3.01 | 2.71 |  | 0.0 | <LOD | <LOD | <LOD | <LOD |
| Ergotaminine | 0.0 | <LOD | <LOD | <LOD | <LOD |  | 55.6 | 0.46 | 0.81 | 0.60 | 0.54 |  | 0.0 | <LOD | <LOD | <LOD | <LOD |
| Fallacinol | 0.0 | <LOD | <LOD | <LOD | <LOD |  | 33.3 | 1.65 | 13.3 | 6.98 | 6.44 |  | 10.5 | 6.98 | 8.88 | 7.93 | 7.93 |
| Fellutanine A | 60.9 | 12.1 | 39.6 | 22.3 | 19.6 |  | 100 | 13.1 | 49.2 | 33.2 | 33.7 |  | 0.0 | <LOD | <LOD | <LOD | <LOD |
| Flavoglaucin | 8.7 | 7.00 | 8.74 | 7.87 | 7.87 |  | 100 | 7.10 | 3477 | 653 | 68.9 |  | 84.2 | 5.35 | 6064 | 554 | 20.2 |
| Ilicicolin A | 13.0 | 0.21 | 0.74 | 0.50 | 0.56 |  | 5.6 | 0.48 | 0.48 | 0.48 | 0.48 |  | 0.0 | <LOD | <LOD | <LOD | <LOD |
| Ilicicolin B | 4.3 | 0.65 | 0.65 | 0.65 | 0.65 |  | 0.0 | <LOD | <LOD | <LOD | <LOD |  | 0.0 | <LOD | <LOD | <LOD | <LOD |
| Ilicicolin E | 0.0 | <LOD | <LOD | <LOD | <LOD |  | 5.6 | 0.52 | 0.52 | 0.52 | 0.52 |  | 0.0 | <LOD | <LOD | <LOD | <LOD |
| Ilicolin C | 0.0 | <LOD | <LOD | <LOD | <LOD |  | 5.6 | 2.86 | 2.86 | 2.86 | 2.86 |  | 0.0 | <LOD | <LOD | <LOD | <LOD |
| Infectopyron | 0.0 | <LOD | <LOD | <LOD | <LOD |  | 88.9 | 51.1 | 158 | 93.7 | 92.3 |  | 0.0 | <LOD | <LOD | <LOD | <LOD |
| Iso-RHDM^c^ | 0.0 | <LOD | <LOD | <LOD | <LOD |  | 38.9 | 0.53 | 2.52 | 1.37 | 1.22 |  | 15.8 | 0.66 | 2.10 | 1.17 | 0.76 |
| Kojic acid | 100 | 40.3 | 174 | 102 | 95.5 |  | 100 | 155 | 924 | 511 | 487 |  | 94.7 | 96.4 | 586 | 237 | 201 |
| LL-Z 1272e | 0.0 | <LOD | <LOD | <LOD | <LOD |  | 5.6 | 0.20 | 0.20 | 0.20 | 0.20 |  | 0.0 | <LOD | <LOD | <LOD | <LOD |
| Macrosporin | 4.3 | 0.65 | 0.65 | 0.65 | 0.65 |  | 88.9 | 1.00 | 1.74 | 1.28 | 1.18 |  | 10.5 | 2.34 | 2.83 | 2.58 | 2.58 |
| Monocerin | 0.0 | <LOD | <LOD | <LOD | <LOD |  | 83.3 | 0.67 | 44.3 | 6.29 | 2.59 |  | 36.8 | 0.19 | 48.0 | 9.78 | 4.62 |
| Mycophenolic acid | 0.0 | <LOD | <LOD | <LOD | <LOD |  | 22.2 | 1.48 | 9.12 | 5.83 | 6.35 |  | 5.3 | 29.1 | 29.1 | 29.1 | 29.1 |
| N-Benzoyl-PHNAL^c^ | 26.1 | 0.55 | 2.42 | 1.01 | 0.75 |  | 100 | 0.88 | 44.5 | 6.15 | 2.52 |  | 42.1 | 0.84 | 12.4 | 3.88 | 2.55 |
| Neoechinulin A | 0.0 | <LOD | <LOD | <LOD | <LOD |  | 72.2 | 1.86 | 242 | 68.3 | 35.7 |  | 21.1 | 8.75 | 790 | 285 | 171 |
| Nidurufin | 0.0 | <LOD | <LOD | <LOD | <LOD |  | 11.1 | 0.54 | 1.56 | 1.05 | 1.05 |  | 0.0 | <LOD | <LOD | <LOD | <LOD |
| Norsolorinic acid | 4.3 | 1.02 | 1.02 | 1.02 | 1.02 |  | 33.3 | 0.97 | 3.92 | 1.83 | 1.43 |  | 0.0 | <LOD | <LOD | <LOD | <LOD |
| O-MethylSTER | 0.0 | <LOD | <LOD | <LOD | <LOD |  | 55.6 | 0.34 | 3.78 | 0.95 | 0.62 |  | 0.0 | <LOD | <LOD | <LOD | <LOD |
| Pestalotin | 0.0 | <LOD | <LOD | <LOD | <LOD |  | 44.4 | 2.07 | 7.43 | 4.32 | 3.86 |  | 21.1 | 1.85 | 5.61 | 3.63 | 3.52 |
| Phenopyrrozin | 91.3 | 2.21 | 30.3 | 7.73 | 4.31 |  | 11.1 | 1.69 | 2.07 | 1.88 | 1.88 |  | 0.0 | <LOD | <LOD | <LOD | <LOD |
| Pinselin | 4.3 | 3.72 | 3.72 | 3.72 | 3.72 |  | 27.8 | 3.97 | 10.8 | 7.00 | 5.38 |  | 0.0 | <LOD | <LOD | <LOD | <LOD |
| Purpactin A | 0.0 | <LOD | <LOD | <LOD | <LOD |  | 5.6 | 1.66 | 1.66 | 1.66 | 1.66 |  | 0.0 | <LOD | <LOD | <LOD | <LOD |
| Questiomycin A | 0.0 | <LOD | <LOD | <LOD | <LOD |  | 83.3 | 2.20 | 26.9 | 10.7 | 6.92 |  | 63.2 | 3.14 | 22.4 | 12.8 | 12.3 |

**Supplementary Table 2.** Continued.

| Metabolites | Garri (*n*=23) | | | | |  | *Granola* (*n*=18) | | | | |  | Popcorn (*n*=19) | | | | |
| --- | --- | --- | --- | --- | --- | --- | --- | --- | --- | --- | --- | --- | --- | --- | --- | --- | --- |
|  | %P^a^ | Min | Max | Mean | Median |  | %P^a^ | Min | Max | Mean | Median |  | %P^a^ | Min | Max | Mean | Median |
| Quinolactacin A | 0.0 | <LOD | <LOD | <LOD | <LOD |  | 72.2 | 0.08 | 71.2 | 19.7 | 1.07 |  | 21.1 | 0.16 | 0.55 | 0.30 | 0.25 |
| Rugulovasine A | 0.0 | <LOD | <LOD | <LOD | <LOD |  | 33.3 | 12.5 | 75.3 | 40.7 | 31.9 |  | 10.5 | 10.9 | 39.8 | 25.4 | 25.4 |
| Rugulusovin | 82.6 | 6.11 | 42.6 | 23.1 | 23.0 |  | 77.8 | 2.05 | 30.9 | 8.76 | 5.41 |  | 0.0 | <LOD | <LOD | <LOD | <LOD |
| Secalonic acid D | 0.0 | <LOD | <LOD | <LOD | <LOD |  | 33.3 | 3.99 | 5.93 | 4.79 | 4.43 |  | 0.0 | <LOD | <LOD | <LOD | <LOD |
| Skyrin | 0.0 | <LOD | <LOD | <LOD | <LOD |  | 33.3 | 4.87 | 44.2 | 17.7 | 7.81 |  | 5.3 | 5.72 | 5.72 | 5.72 | 5.72 |
| Sterigmatocystin | 0.0 | <LOD | <LOD | <LOD | <LOD |  | 33.3 | 0.46 | 1.37 | 0.84 | 0.87 |  | 0.0 | <LOD | <LOD | <LOD | <LOD |
| Tentoxin | 0.0 | <LOD | <LOD | <LOD | <LOD |  | 83.3 | 0.55 | 2.78 | 1.57 | 1.53 |  | 0.0 | <LOD | <LOD | <LOD | <LOD |
| Terphenyllin | 0.0 | <LOD | <LOD | <LOD | <LOD |  | 11.1 | 16.3 | 17.5 | 16.9 | 16.9 |  | 0.0 | <LOD | <LOD | <LOD | <LOD |
| Tryptophol | 95.7 | 34.9 | 2880 | 394 | 203 |  | 100 | 69.6 | 190 | 123.1 | 126 |  | 68.4 | 9.79 | 95.6 | 44.5 | 41.5 |
| Versicolorin A | 0.0 | <LOD | <LOD | <LOD | <LOD |  | 27.8 | 0.89 | 3.27 | 1.75 | 1.50 |  | 0.0 | <LOD | <LOD | <LOD | <LOD |
| Versicolorin C | 0.0 | <LOD | <LOD | <LOD | <LOD |  | 61.1 | 0.95 | 37.2 | 9.10 | 4.66 |  | 0.0 | <LOD | <LOD | <LOD | <LOD |

^a^Percentage of contaminated samples.

^b^Values denote peak areas due to lack of standard.

^c^3-Nitropropionic acid; 7-Hydroxypestalotin; Iso-Rhodoptilometrin; N-Benzoyl-Phenylalanine.
