# Supplementary material for: Fungal Diversity and Mycotoxins in Low Moisture Content Ready-To-Eat Foods in Nigeria
Source: Front Microbiol. 2020 Apr 9;11:615. doi: 10.3389/fmicb.2020.00615 (PMC7161469; doi:10.3389/fmicb.2020.00615)
Supplement: Supplementary file 2 [file Presentation_1.PPTX]

## Slide 1
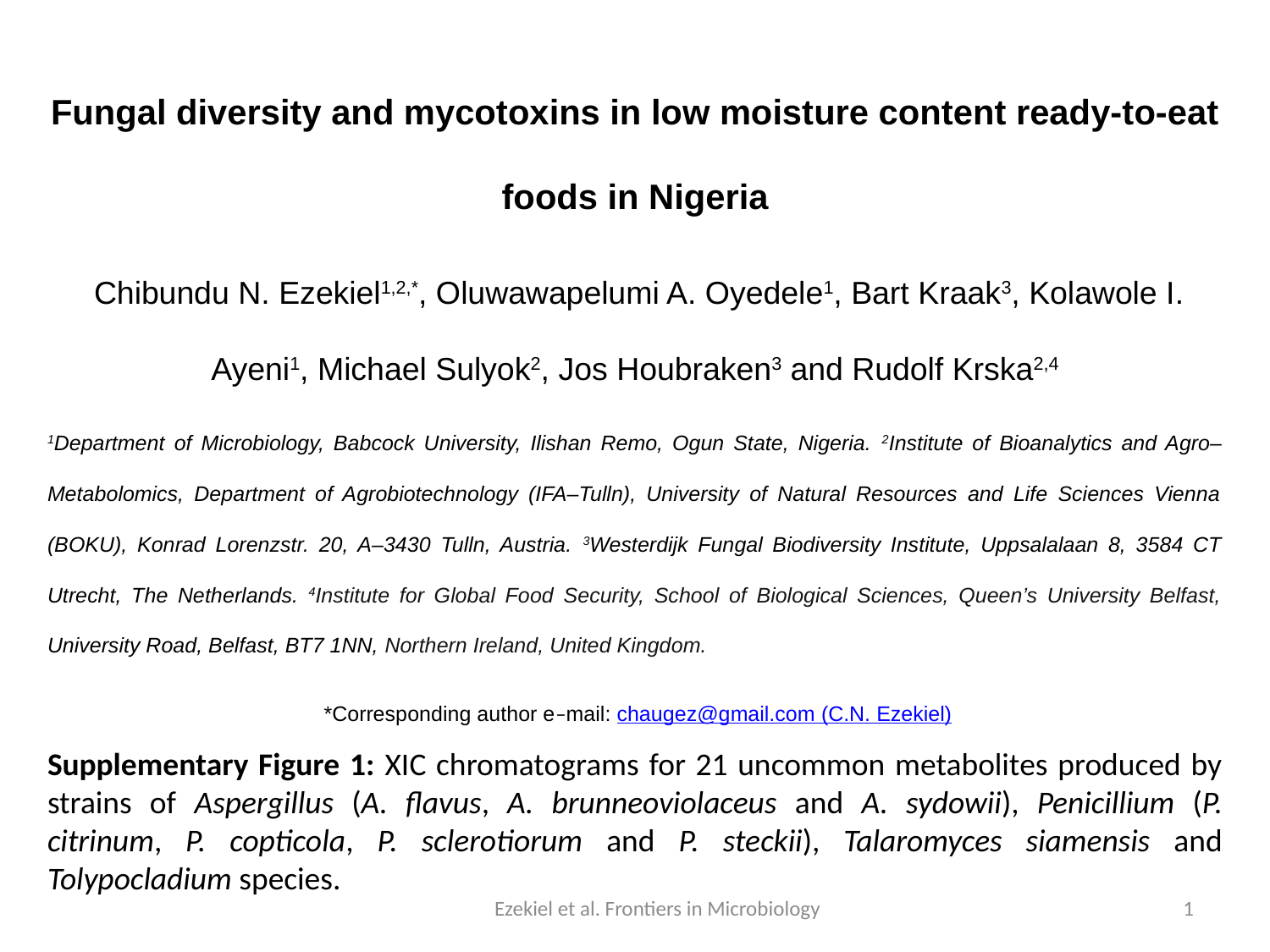

Fungal diversity and mycotoxins in low moisture content ready-to-eat foods in Nigeria
 Chibundu N. Ezekiel1,2,*, Oluwawapelumi A. Oyedele1, Bart Kraak3, Kolawole I. Ayeni1, Michael Sulyok2, Jos Houbraken3 and Rudolf Krska2,4
1Department of Microbiology, Babcock University, Ilishan Remo, Ogun State, Nigeria. 2Institute of Bioanalytics and Agro–Metabolomics, Department of Agrobiotechnology (IFA–Tulln), University of Natural Resources and Life Sciences Vienna (BOKU), Konrad Lorenzstr. 20, A–3430 Tulln, Austria. 3Westerdijk Fungal Biodiversity Institute, Uppsalalaan 8, 3584 CT Utrecht, The Netherlands. 4Institute for Global Food Security, School of Biological Sciences, Queen’s University Belfast, University Road, Belfast, BT7 1NN, Northern Ireland, United Kingdom.
 *Corresponding author e–mail: chaugez@gmail.com (C.N. Ezekiel)
Supplementary Figure 1: XIC chromatograms for 21 uncommon metabolites produced by strains of Aspergillus (A. flavus, A. brunneoviolaceus and A. sydowii), Penicillium (P. citrinum, P. copticola, P. sclerotiorum and P. steckii), Talaromyces siamensis and Tolypocladium species.
Ezekiel et al. Frontiers in Microbiology
1

## Slide 2
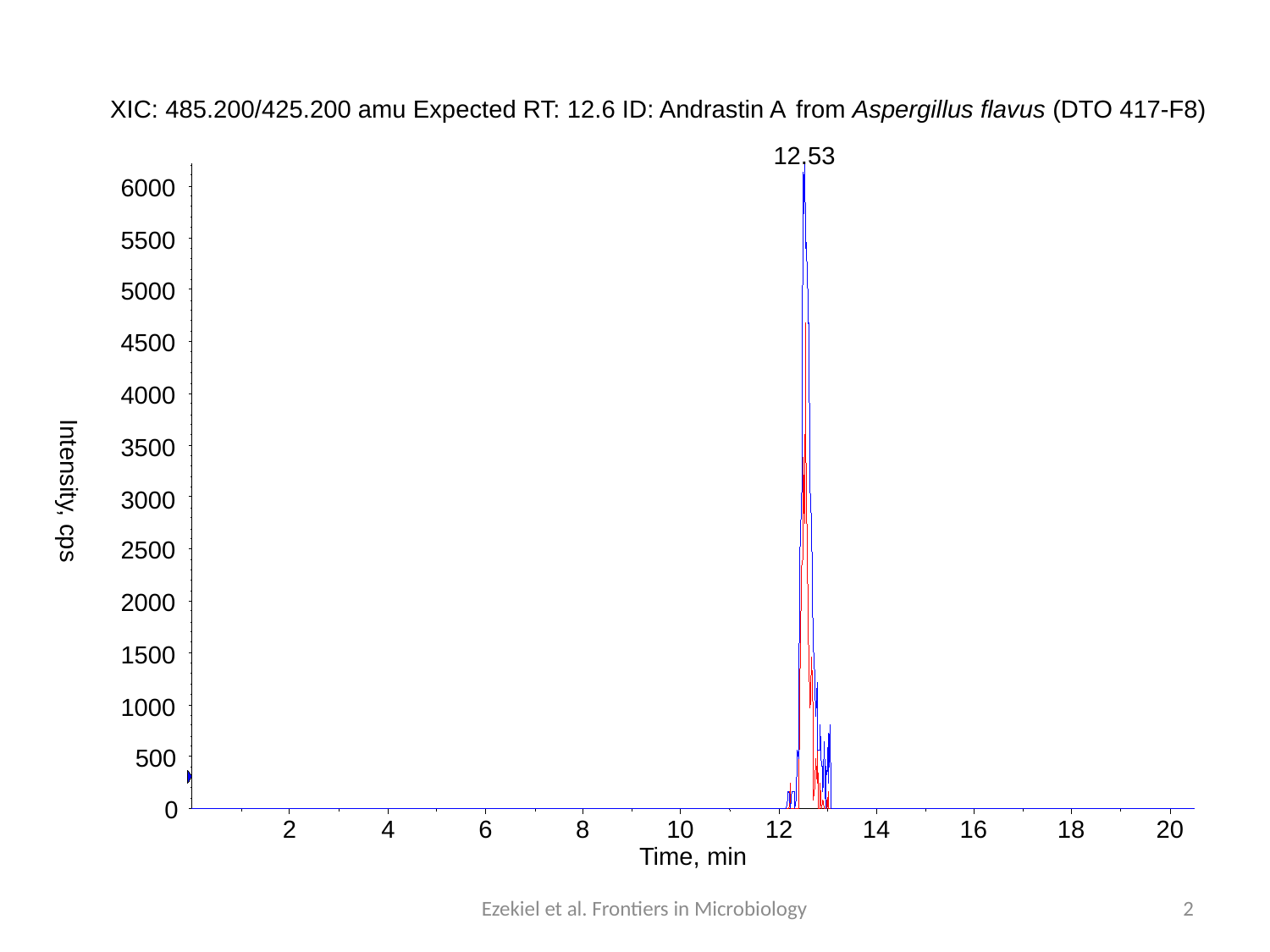

XIC: 485.200/425.200 amu Expected RT: 12.6 ID: Andrastin A from Aspergillus flavus (DTO 417-F8)
12.53
6000
5500
5000
4500
4000
3500
Intensity, cps
3000
2500
2000
1500
1000
500
0
2
4
6
8
10
12
14
16
18
20
Time, min
Ezekiel et al. Frontiers in Microbiology
2

## Slide 3
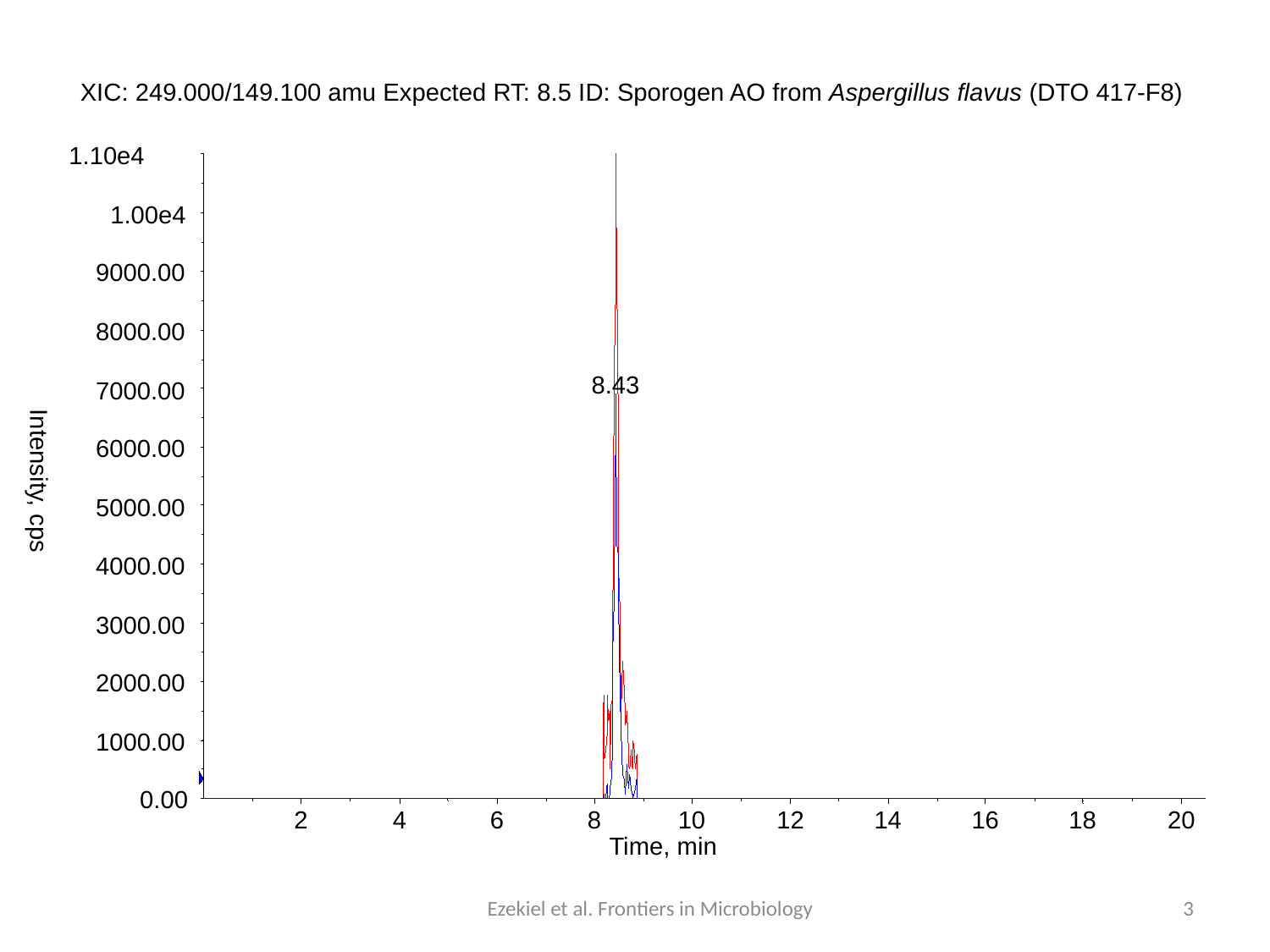

XIC: 249.000/149.100 amu Expected RT: 8.5 ID: Sporogen AO from Aspergillus flavus (DTO 417-F8)
1.10e4
1.00e4
9000.00
8000.00
8.43
7000.00
6000.00
Intensity, cps
5000.00
4000.00
3000.00
2000.00
1000.00
0.00
2
4
6
8
10
12
14
16
18
20
Time, min
Ezekiel et al. Frontiers in Microbiology
3

## Slide 4
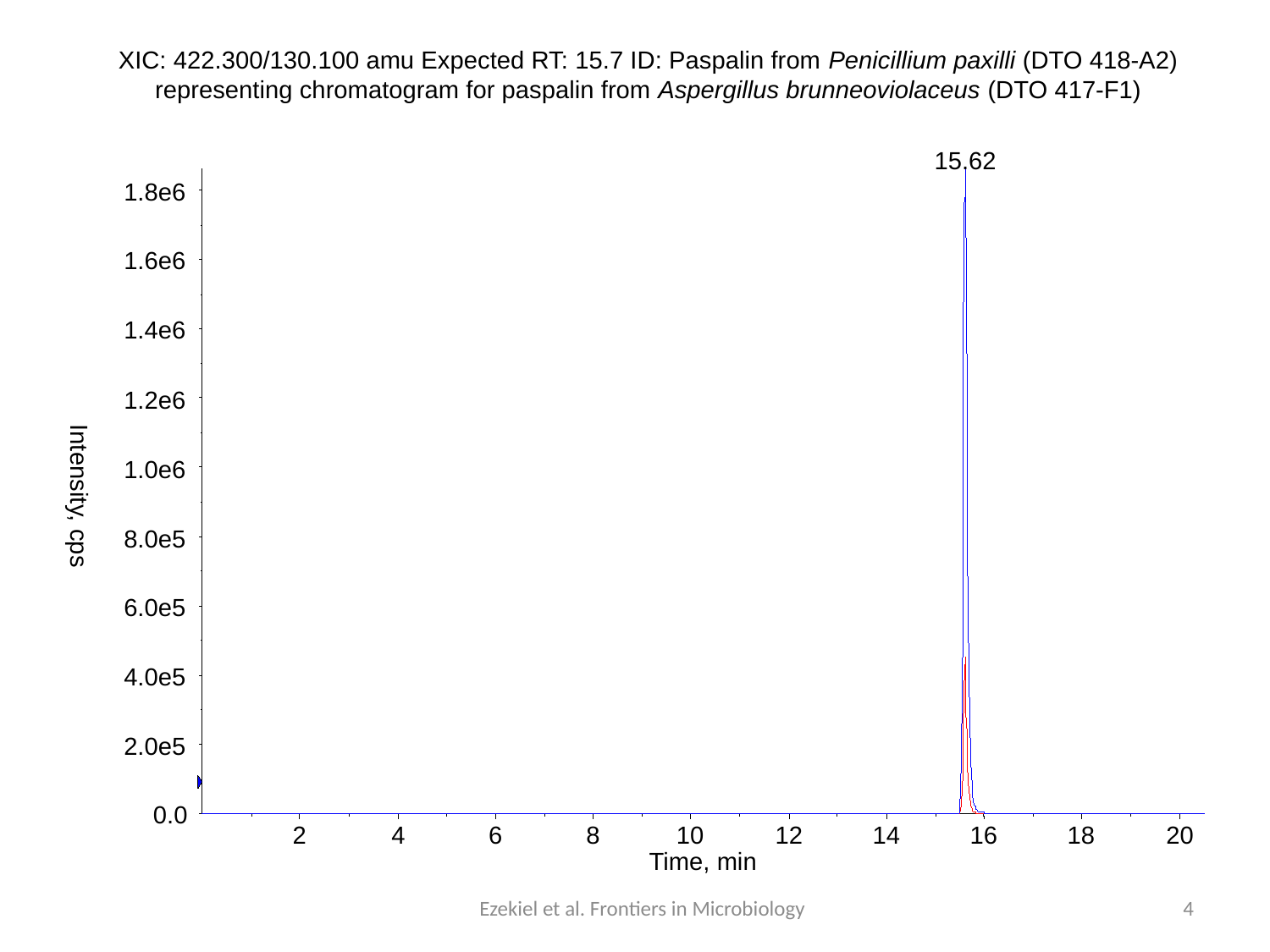

XIC: 422.300/130.100 amu Expected RT: 15.7 ID: Paspalin from Penicillium paxilli (DTO 418-A2) representing chromatogram for paspalin from Aspergillus brunneoviolaceus (DTO 417-F1)
15.62
1.8e6
1.6e6
1.4e6
1.2e6
1.0e6
Intensity, cps
8.0e5
6.0e5
4.0e5
2.0e5
0.0
2
4
6
8
10
12
14
16
18
20
Time, min
Ezekiel et al. Frontiers in Microbiology
4

## Slide 5
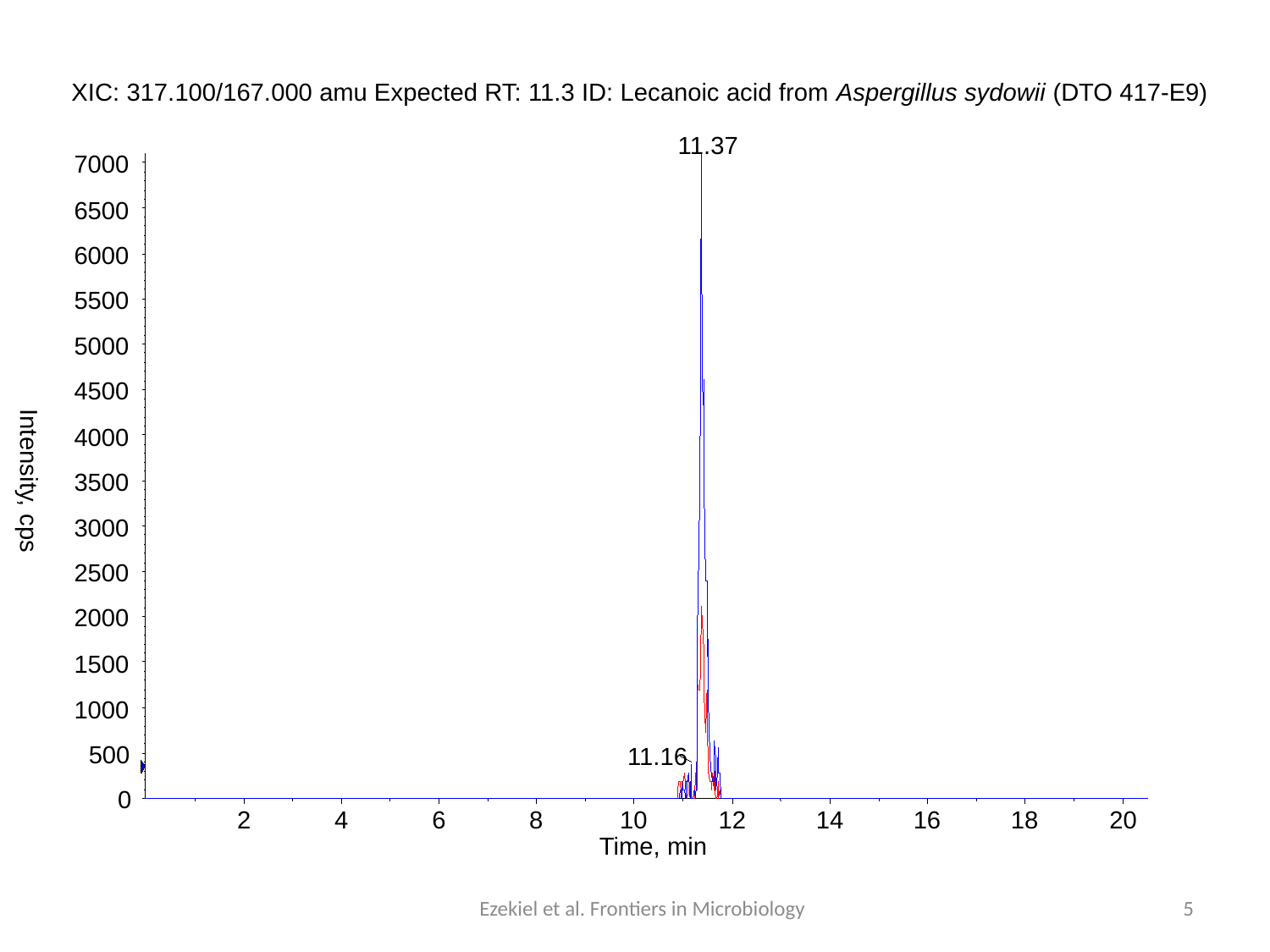

XIC: 317.100/167.000 amu Expected RT: 11.3 ID: Lecanoic acid from Aspergillus sydowii (DTO 417-E9)
11.37
7000
6500
6000
5500
5000
4500
4000
Intensity, cps
3500
3000
2500
2000
1500
1000
500
11.16
0
2
4
6
8
10
12
14
16
18
20
Time, min
Ezekiel et al. Frontiers in Microbiology
5

## Slide 6
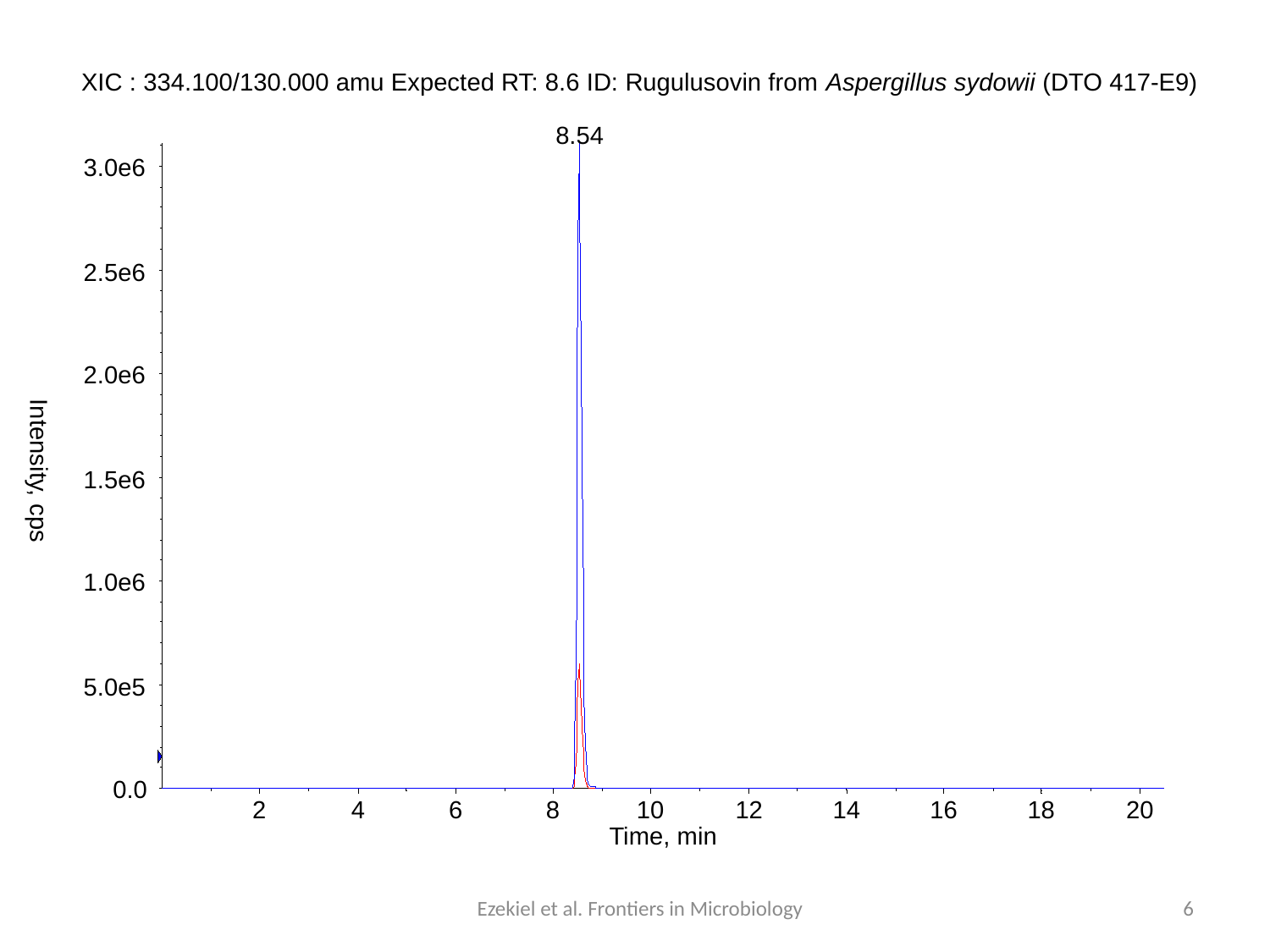

XIC : 334.100/130.000 amu Expected RT: 8.6 ID: Rugulusovin from Aspergillus sydowii (DTO 417-E9)
8.54
3.0e6
2.5e6
2.0e6
Intensity, cps
1.5e6
1.0e6
5.0e5
0.0
2
4
6
8
10
12
14
16
18
20
Time, min
Ezekiel et al. Frontiers in Microbiology
6

## Slide 7
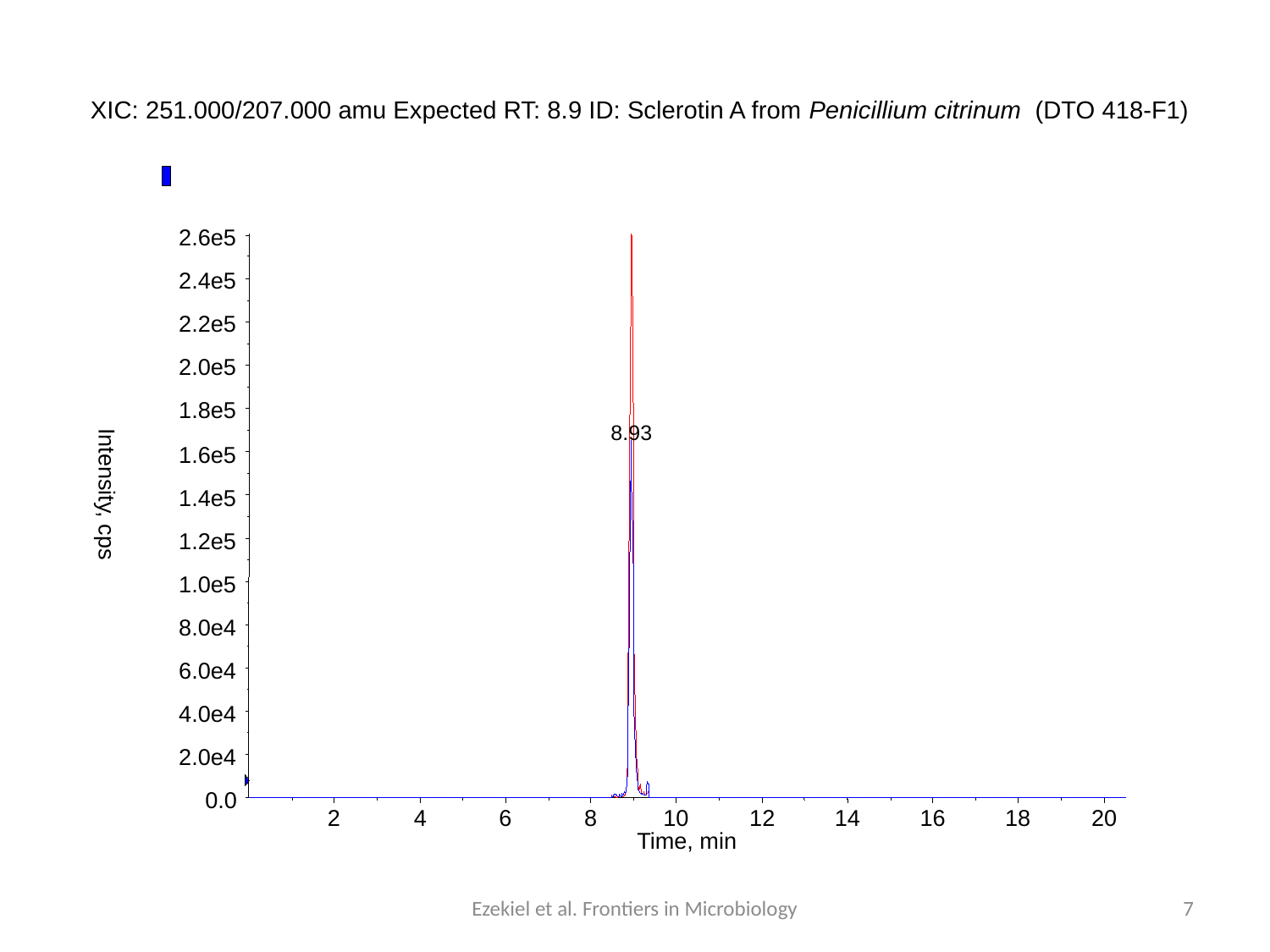

XIC: 251.000/207.000 amu Expected RT: 8.9 ID: Sclerotin A from Penicillium citrinum (DTO 418-F1)
2.6e5
2.4e5
2.2e5
2.0e5
1.8e5
8.93
1.6e5
Intensity, cps
1.4e5
1.2e5
1.0e5
8.0e4
6.0e4
4.0e4
2.0e4
0.0
2
4
6
8
10
12
14
16
18
20
Time, min
Ezekiel et al. Frontiers in Microbiology
7

## Slide 8
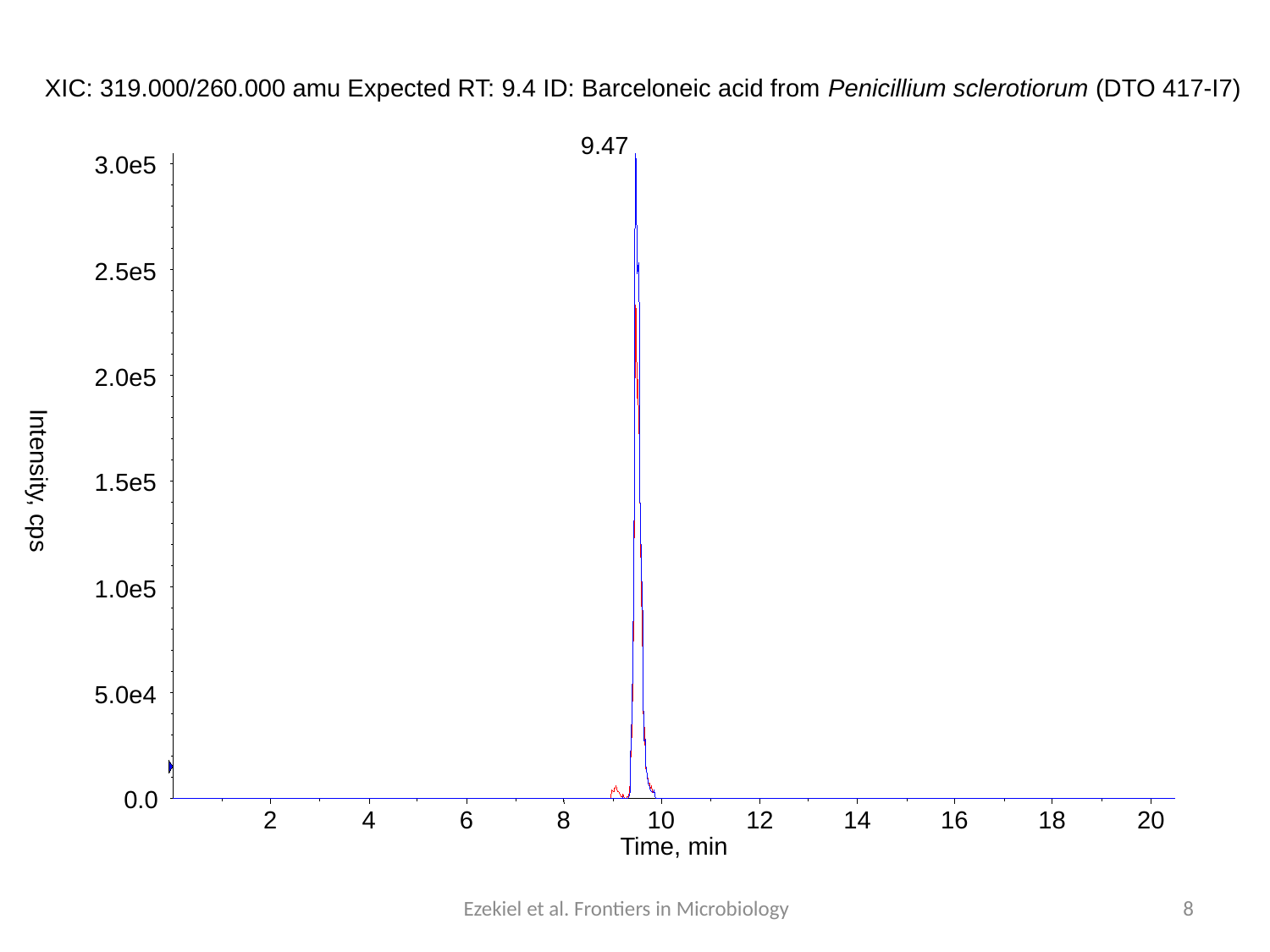

XIC: 319.000/260.000 amu Expected RT: 9.4 ID: Barceloneic acid from Penicillium sclerotiorum (DTO 417-I7)
9.47
3.0e5
2.5e5
2.0e5
Intensity, cps
1.5e5
1.0e5
5.0e4
0.0
2
4
6
8
10
12
14
16
18
20
Time, min
Ezekiel et al. Frontiers in Microbiology
8

## Slide 9
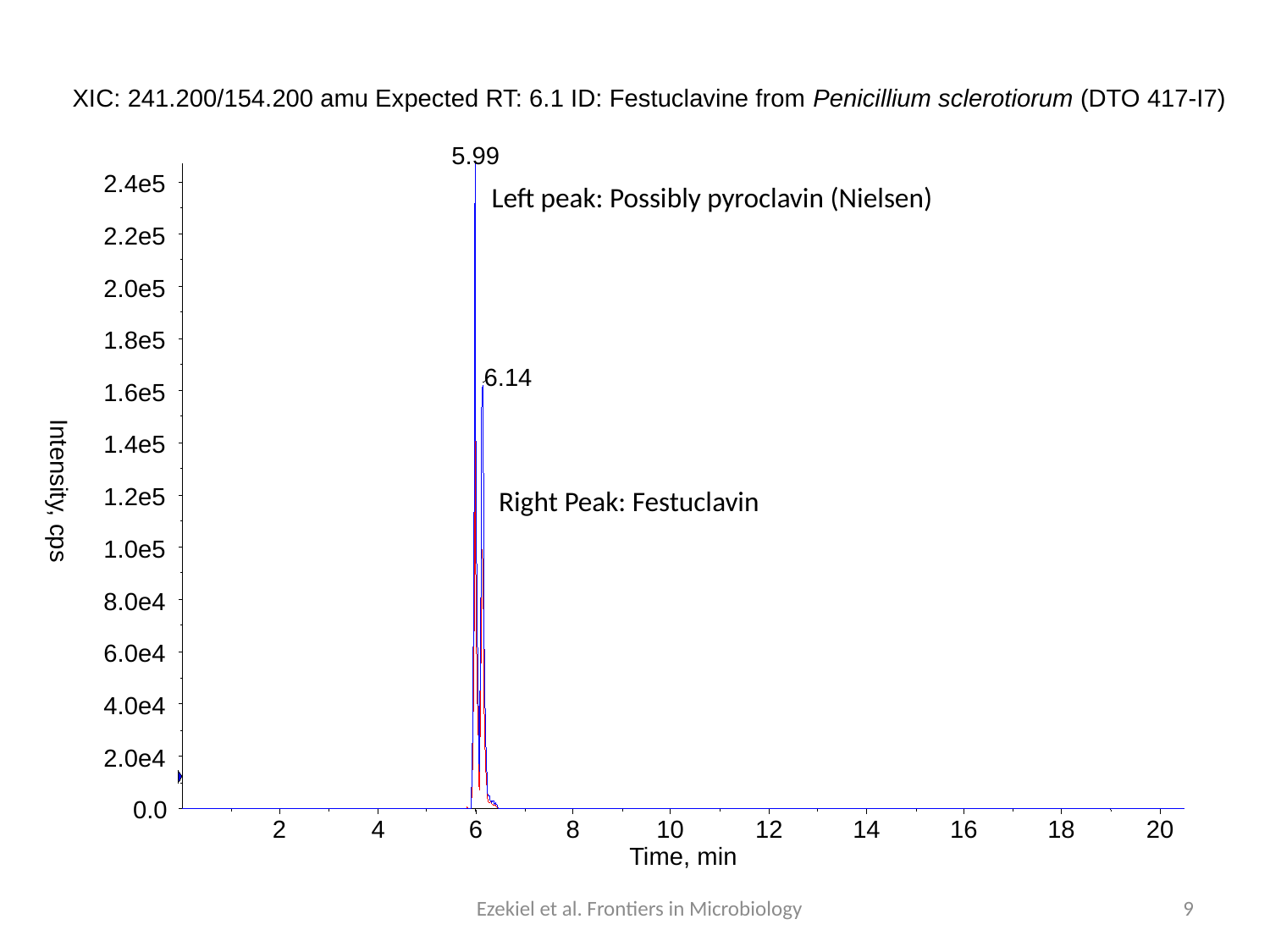

XIC: 241.200/154.200 amu Expected RT: 6.1 ID: Festuclavine from Penicillium sclerotiorum (DTO 417-I7)
5.99
2.4e5
Left peak: Possibly pyroclavin (Nielsen)
2.2e5
2.0e5
1.8e5
6.14
1.6e5
1.4e5
Intensity, cps
Right Peak: Festuclavin
1.2e5
1.0e5
8.0e4
6.0e4
4.0e4
2.0e4
0.0
2
4
6
8
10
12
14
16
18
20
Time, min
Ezekiel et al. Frontiers in Microbiology
9

## Slide 10
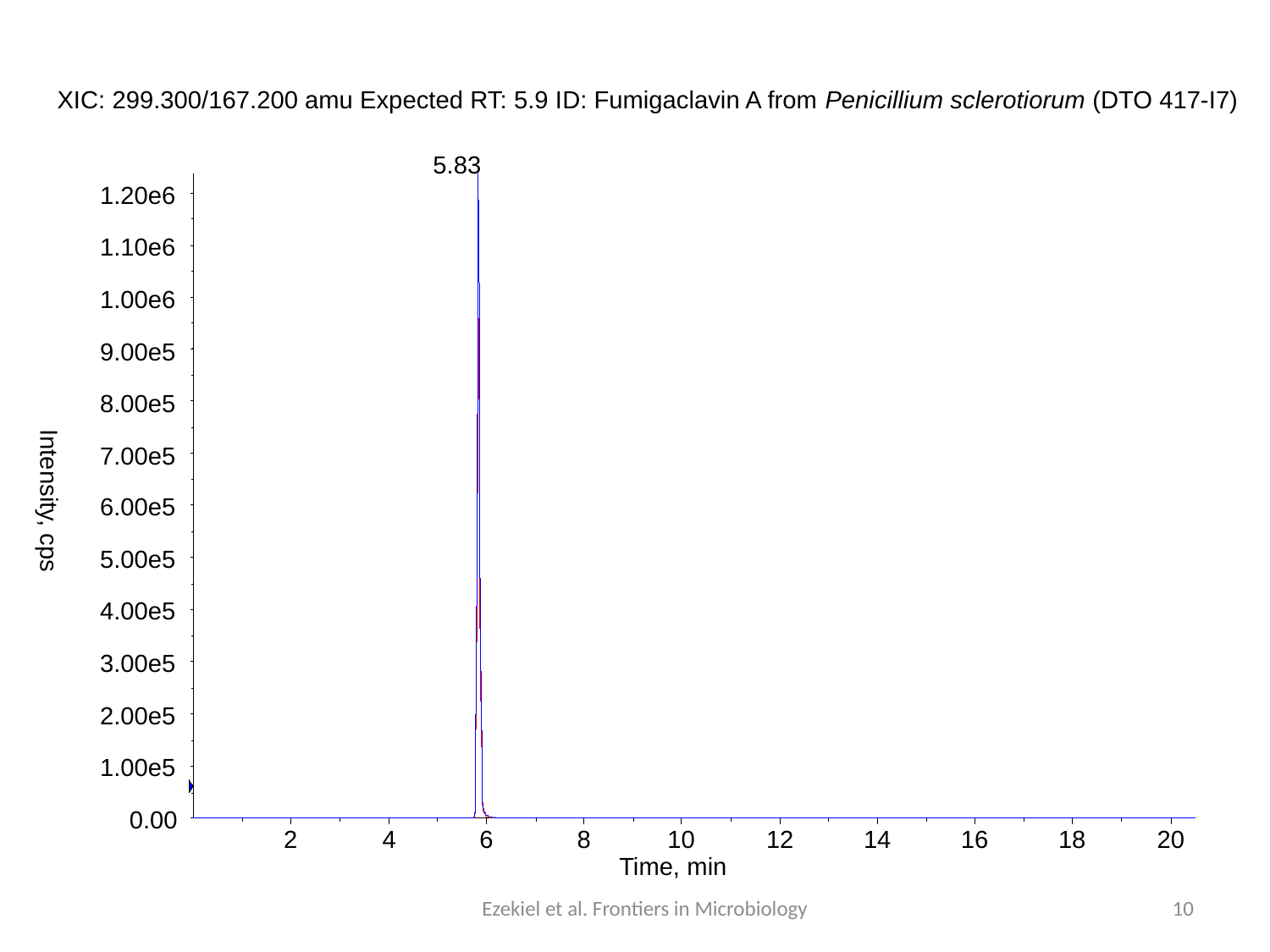

XIC: 299.300/167.200 amu Expected RT: 5.9 ID: Fumigaclavin A from Penicillium sclerotiorum (DTO 417-I7)
5.83
1.20e6
1.10e6
1.00e6
9.00e5
8.00e5
7.00e5
Intensity, cps
6.00e5
5.00e5
4.00e5
3.00e5
2.00e5
1.00e5
0.00
2
4
6
8
10
12
14
16
18
20
Time, min
Ezekiel et al. Frontiers in Microbiology
10

## Slide 11
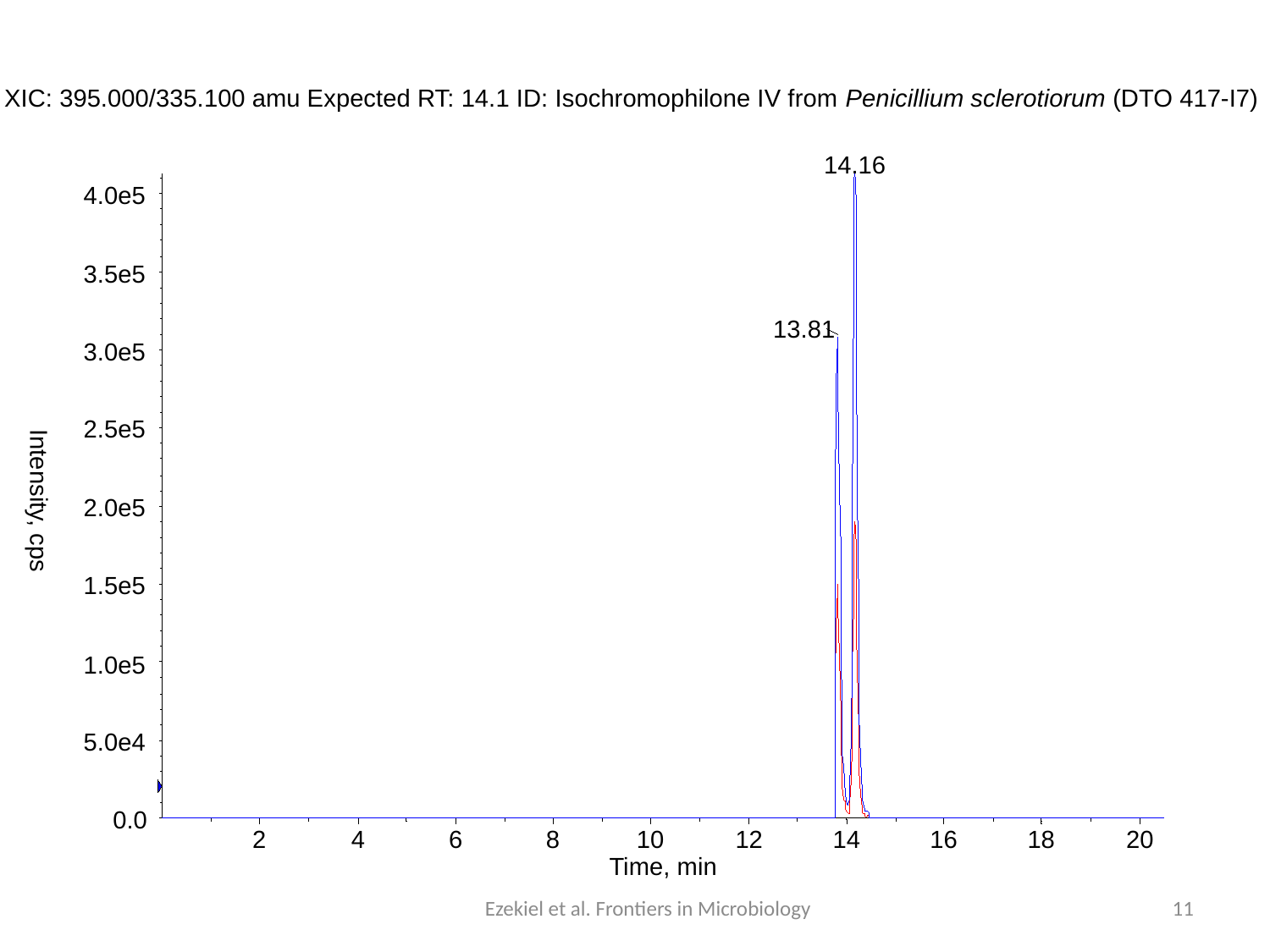

XIC: 395.000/335.100 amu Expected RT: 14.1 ID: Isochromophilone IV from Penicillium sclerotiorum (DTO 417-I7)
14.16
4.0e5
3.5e5
13.81
3.0e5
2.5e5
Intensity, cps
2.0e5
1.5e5
1.0e5
5.0e4
0.0
2
4
6
8
10
12
14
16
18
20
Time, min
Ezekiel et al. Frontiers in Microbiology
11

## Slide 12
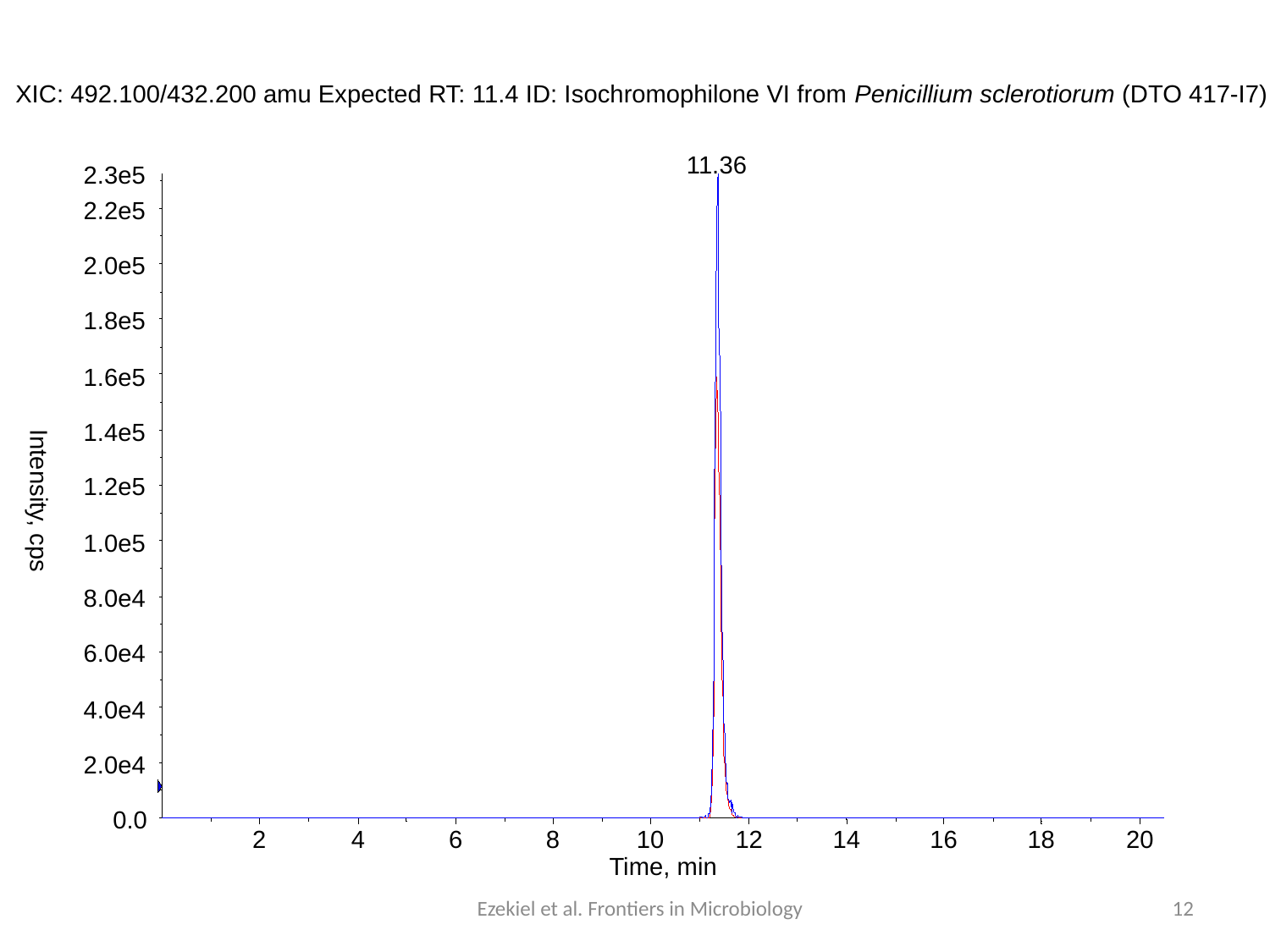

XIC: 492.100/432.200 amu Expected RT: 11.4 ID: Isochromophilone VI from Penicillium sclerotiorum (DTO 417-I7)
11.36
2.3e5
2.2e5
2.0e5
1.8e5
1.6e5
1.4e5
1.2e5
Intensity, cps
1.0e5
8.0e4
6.0e4
4.0e4
2.0e4
0.0
2
4
6
8
10
12
14
16
18
20
Time, min
Ezekiel et al. Frontiers in Microbiology
12

## Slide 13
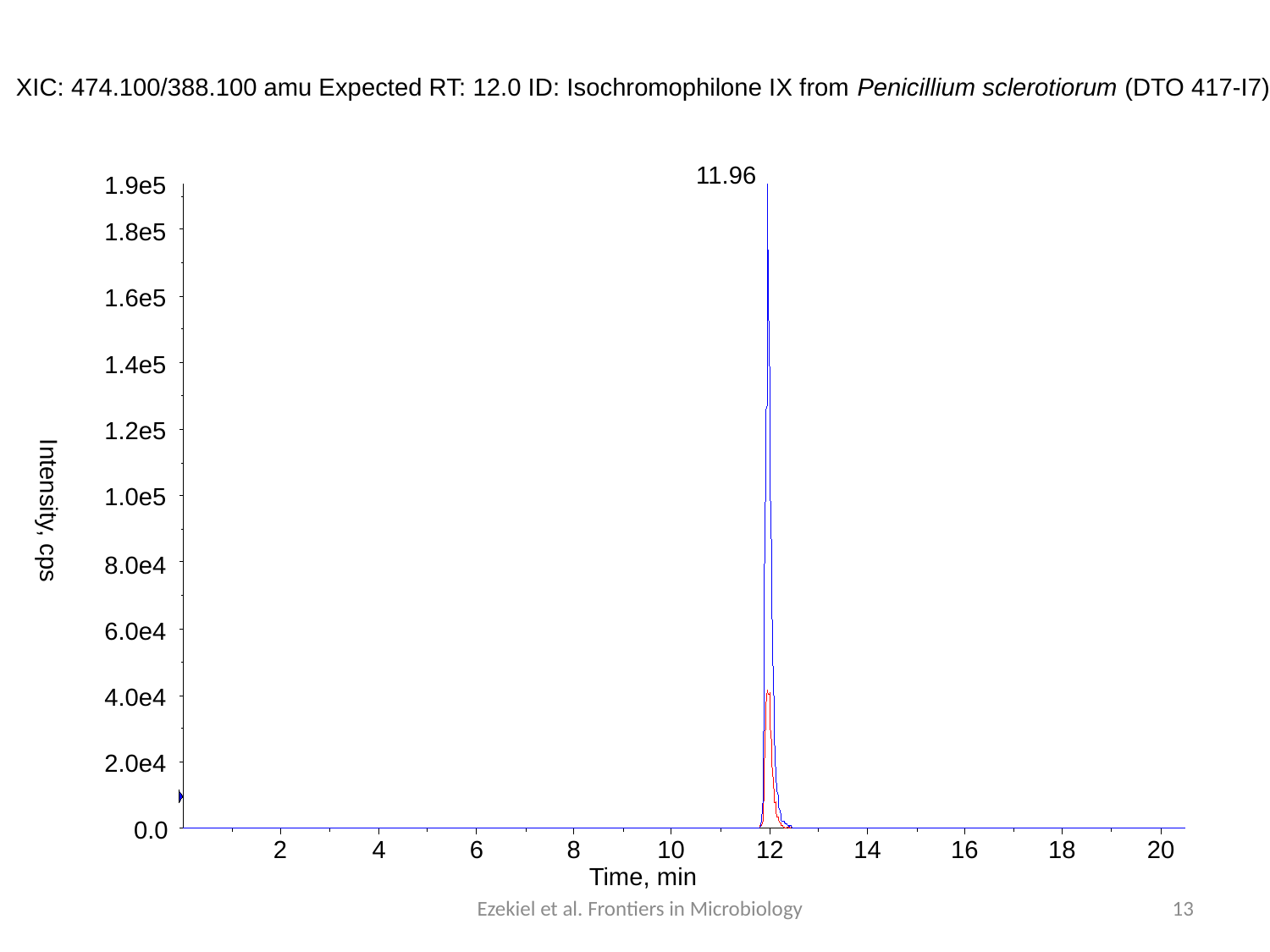

XIC: 474.100/388.100 amu Expected RT: 12.0 ID: Isochromophilone IX from Penicillium sclerotiorum (DTO 417-I7)
11.96
1.9e5
1.8e5
1.6e5
1.4e5
1.2e5
1.0e5
Intensity, cps
8.0e4
6.0e4
4.0e4
2.0e4
0.0
2
4
6
8
10
12
14
16
18
20
Time, min
Ezekiel et al. Frontiers in Microbiology
13

## Slide 14
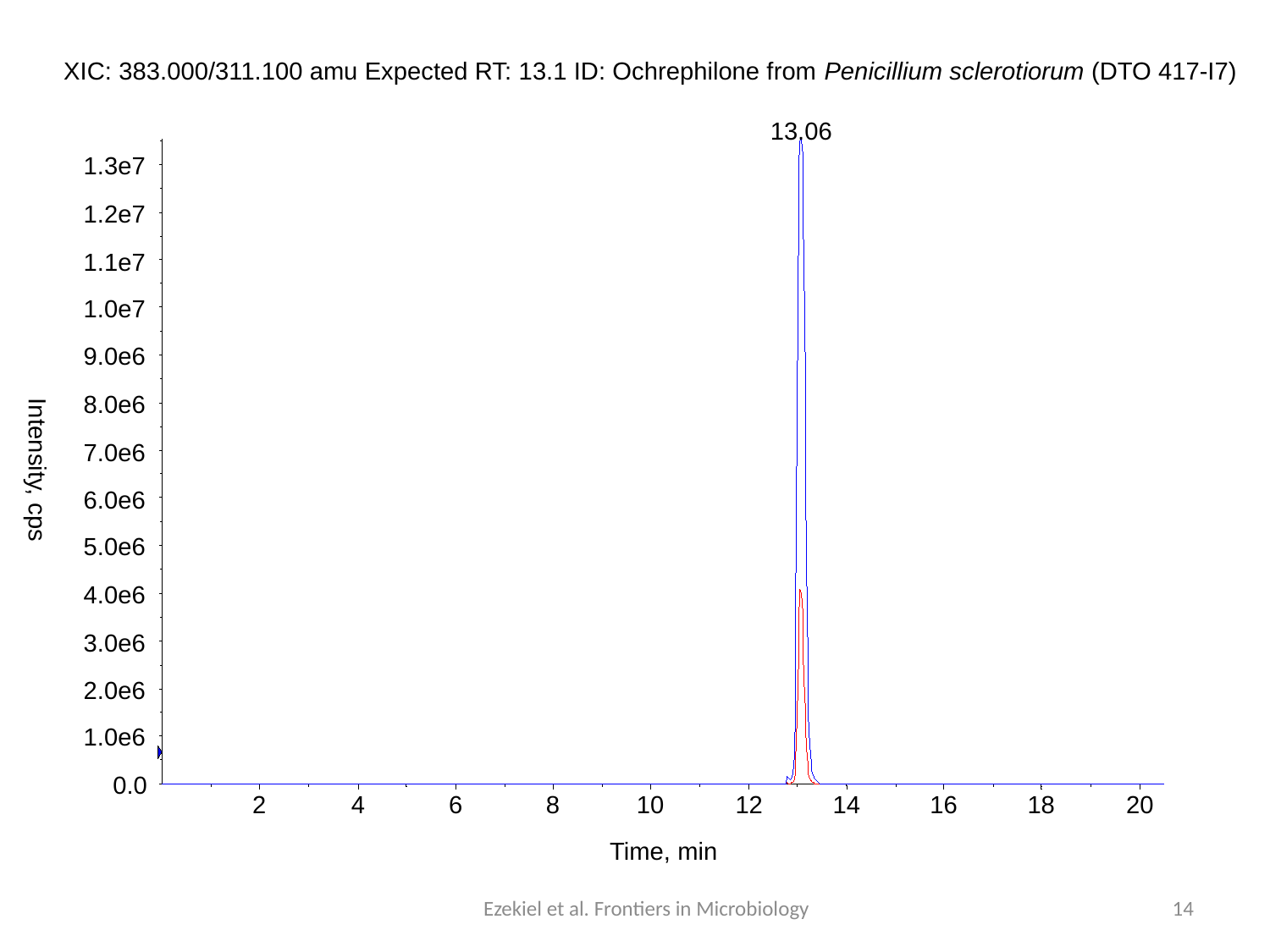

XIC: 383.000/311.100 amu Expected RT: 13.1 ID: Ochrephilone from Penicillium sclerotiorum (DTO 417-I7)
13.06
1.3e7
1.2e7
1.1e7
1.0e7
9.0e6
8.0e6
7.0e6
Intensity, cps
6.0e6
5.0e6
4.0e6
3.0e6
2.0e6
1.0e6
0.0
2
4
6
8
10
12
14
16
18
20
Time, min
Ezekiel et al. Frontiers in Microbiology
14

## Slide 15
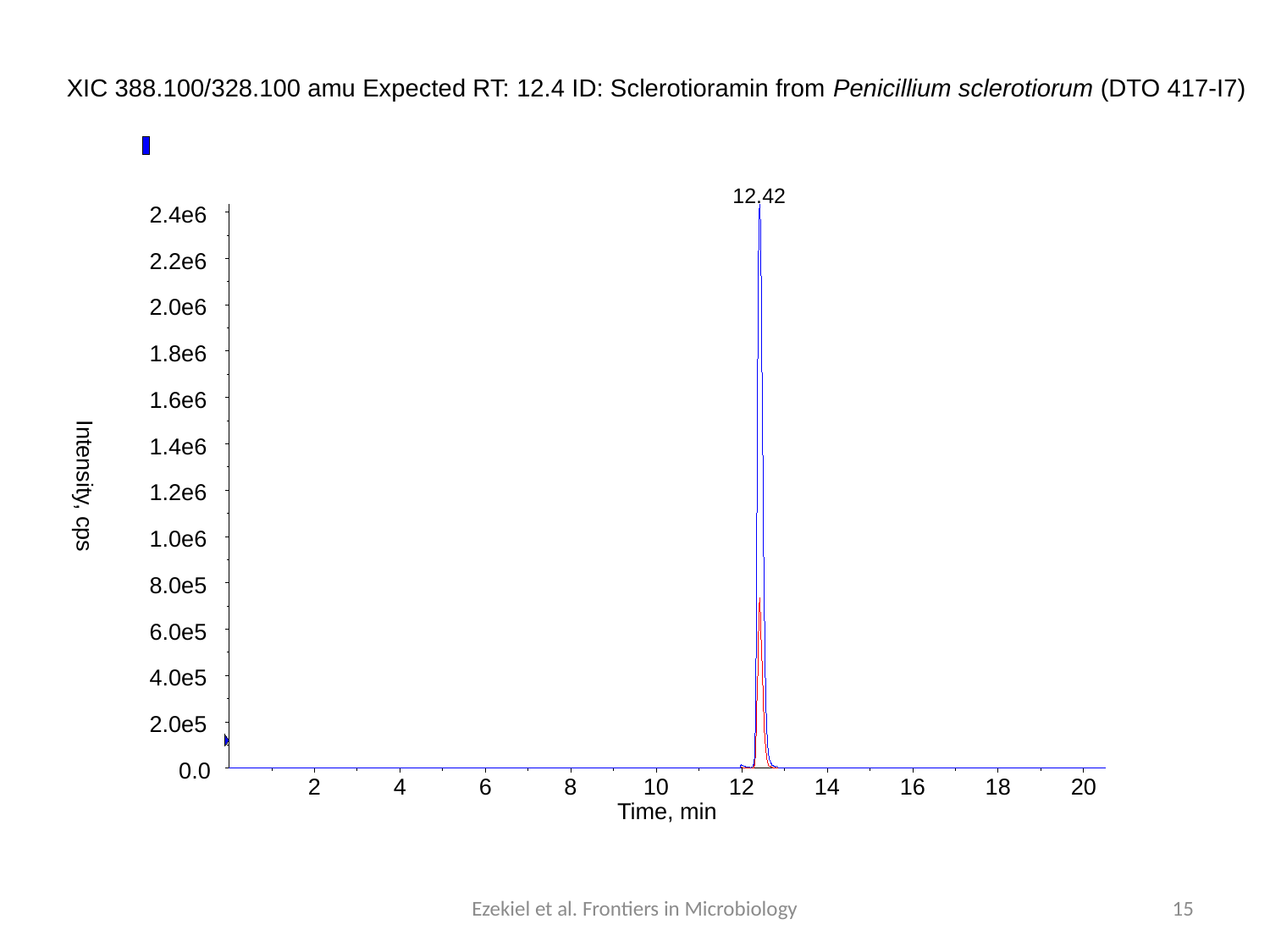

XIC 388.100/328.100 amu Expected RT: 12.4 ID: Sclerotioramin from Penicillium sclerotiorum (DTO 417-I7)
12.42
2.4e6
2.2e6
2.0e6
1.8e6
1.6e6
1.4e6
Intensity, cps
1.2e6
1.0e6
8.0e5
6.0e5
4.0e5
2.0e5
0.0
2
4
6
8
10
12
14
16
18
20
Time, min
Ezekiel et al. Frontiers in Microbiology
15

## Slide 16
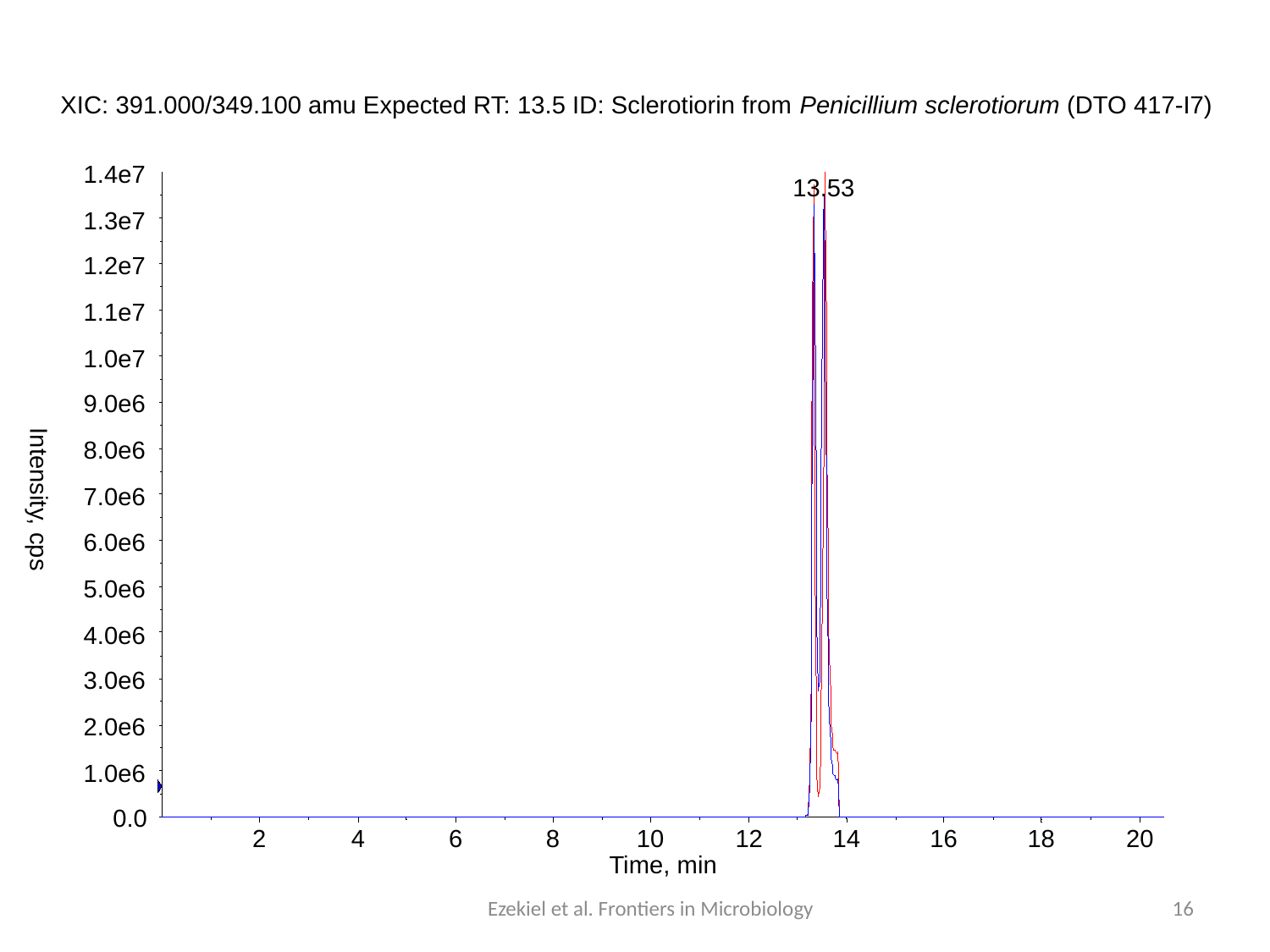

XIC: 391.000/349.100 amu Expected RT: 13.5 ID: Sclerotiorin from Penicillium sclerotiorum (DTO 417-I7)
1.4e7
13.53
1.3e7
1.2e7
1.1e7
1.0e7
9.0e6
8.0e6
Intensity, cps
7.0e6
6.0e6
5.0e6
4.0e6
3.0e6
2.0e6
1.0e6
0.0
2
4
6
8
10
12
14
16
18
20
Time, min
Ezekiel et al. Frontiers in Microbiology
16

## Slide 17
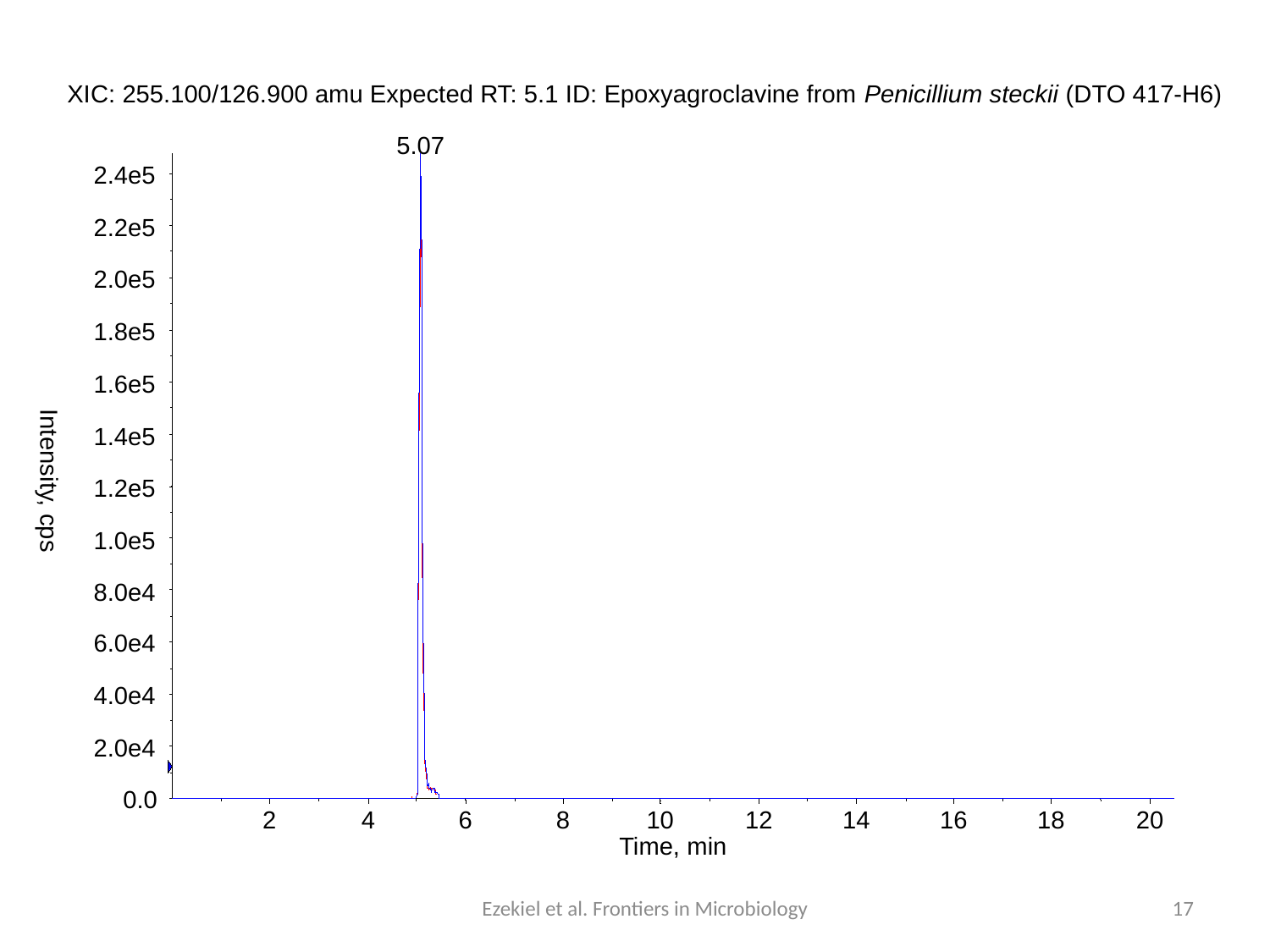

XIC: 255.100/126.900 amu Expected RT: 5.1 ID: Epoxyagroclavine from Penicillium steckii (DTO 417-H6)
5.07
2.4e5
2.2e5
2.0e5
1.8e5
1.6e5
1.4e5
Intensity, cps
1.2e5
1.0e5
8.0e4
6.0e4
4.0e4
2.0e4
0.0
2
4
6
8
10
12
14
16
18
20
Time, min
Ezekiel et al. Frontiers in Microbiology
17

## Slide 18
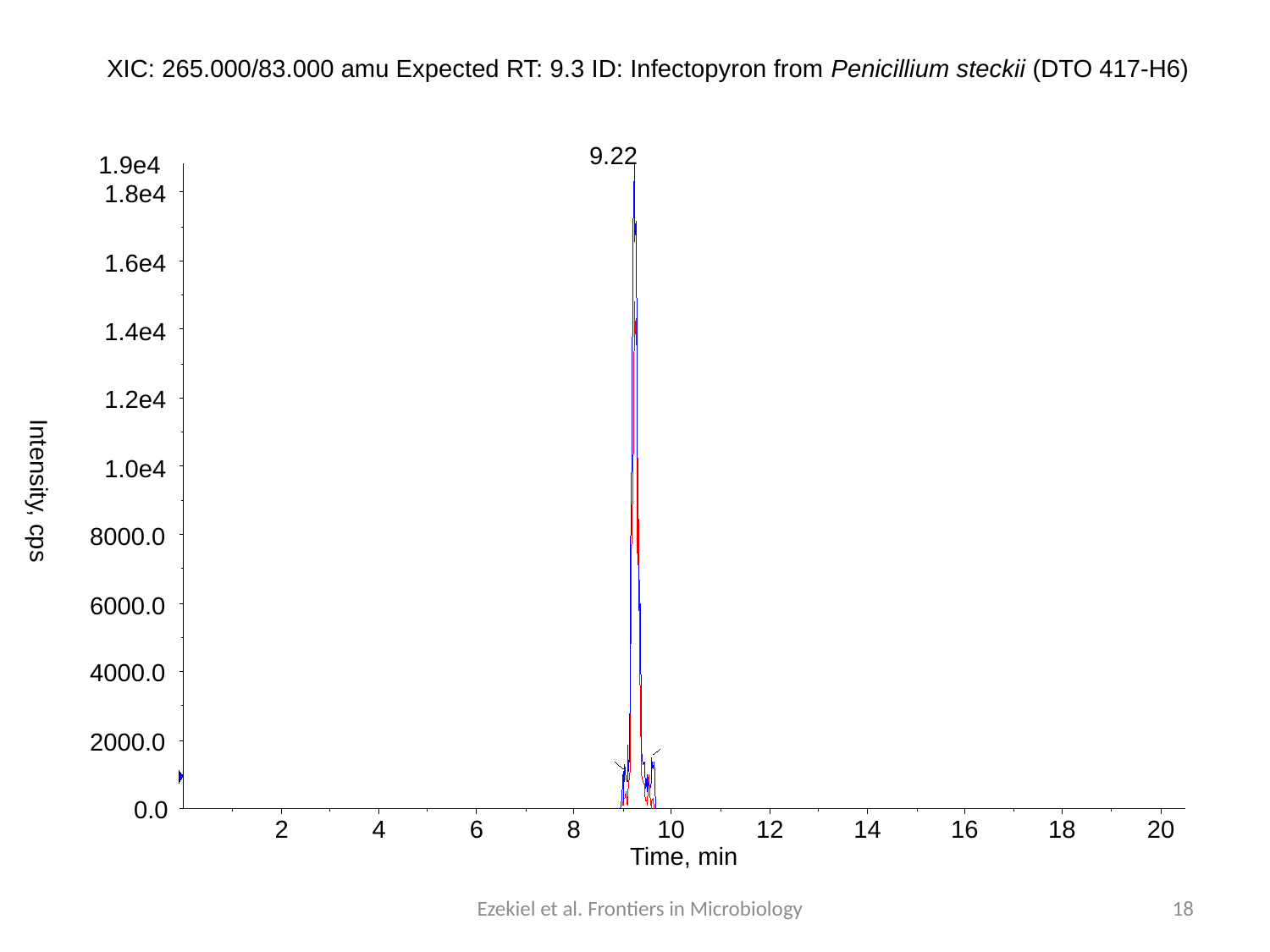

XIC: 265.000/83.000 amu Expected RT: 9.3 ID: Infectopyron from Penicillium steckii (DTO 417-H6)
9.22
1.9e4
1.8e4
1.6e4
1.4e4
1.2e4
1.0e4
Intensity, cps
8000.0
6000.0
4000.0
2000.0
0.0
2
4
6
8
10
12
14
16
18
20
Time, min
Ezekiel et al. Frontiers in Microbiology
18

## Slide 19
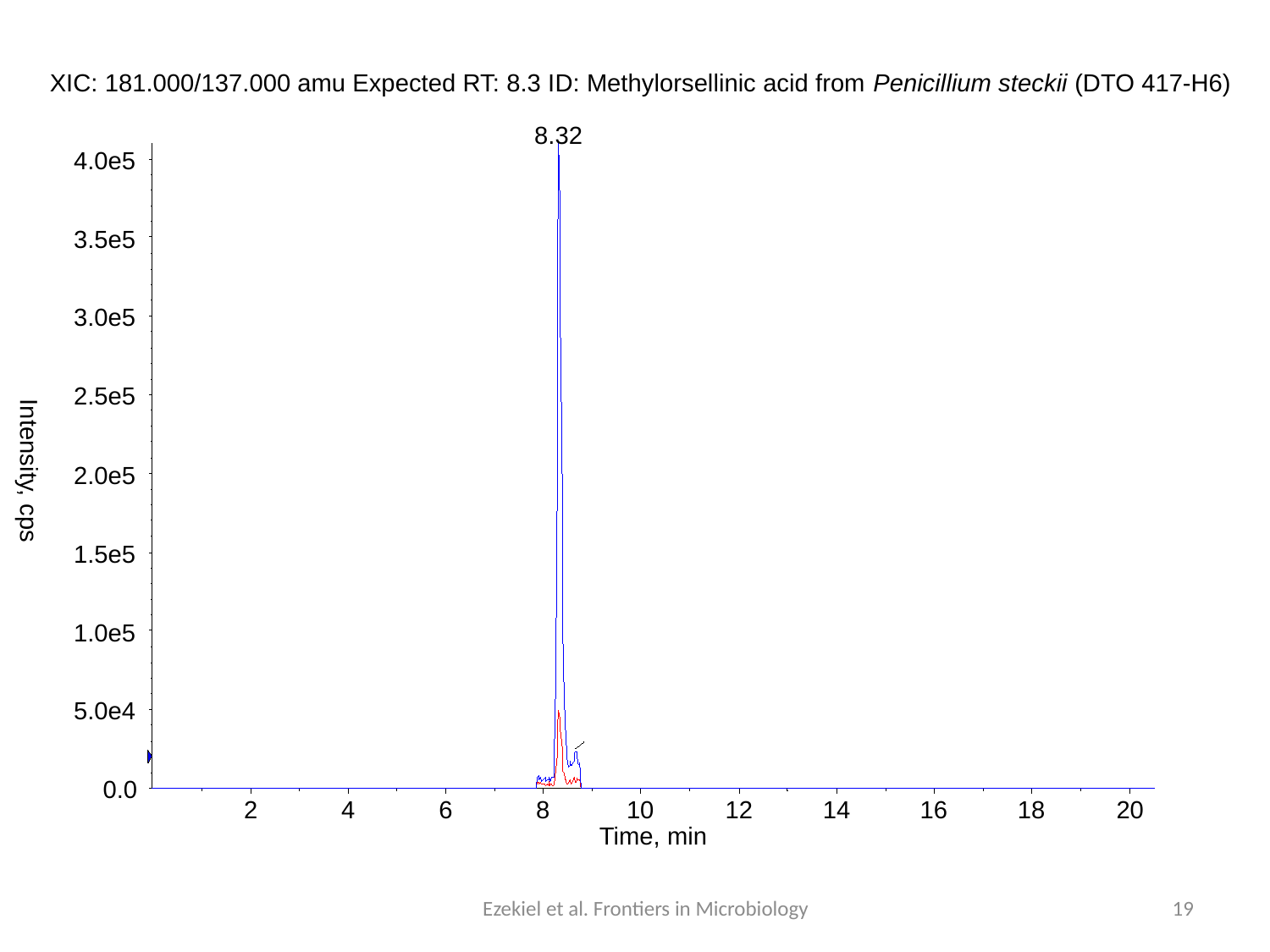

XIC: 181.000/137.000 amu Expected RT: 8.3 ID: Methylorsellinic acid from Penicillium steckii (DTO 417-H6)
8.32
4.0e5
3.5e5
3.0e5
2.5e5
Intensity, cps
2.0e5
1.5e5
1.0e5
5.0e4
0.0
2
4
6
8
10
12
14
16
18
20
Time, min
Ezekiel et al. Frontiers in Microbiology
19

## Slide 20
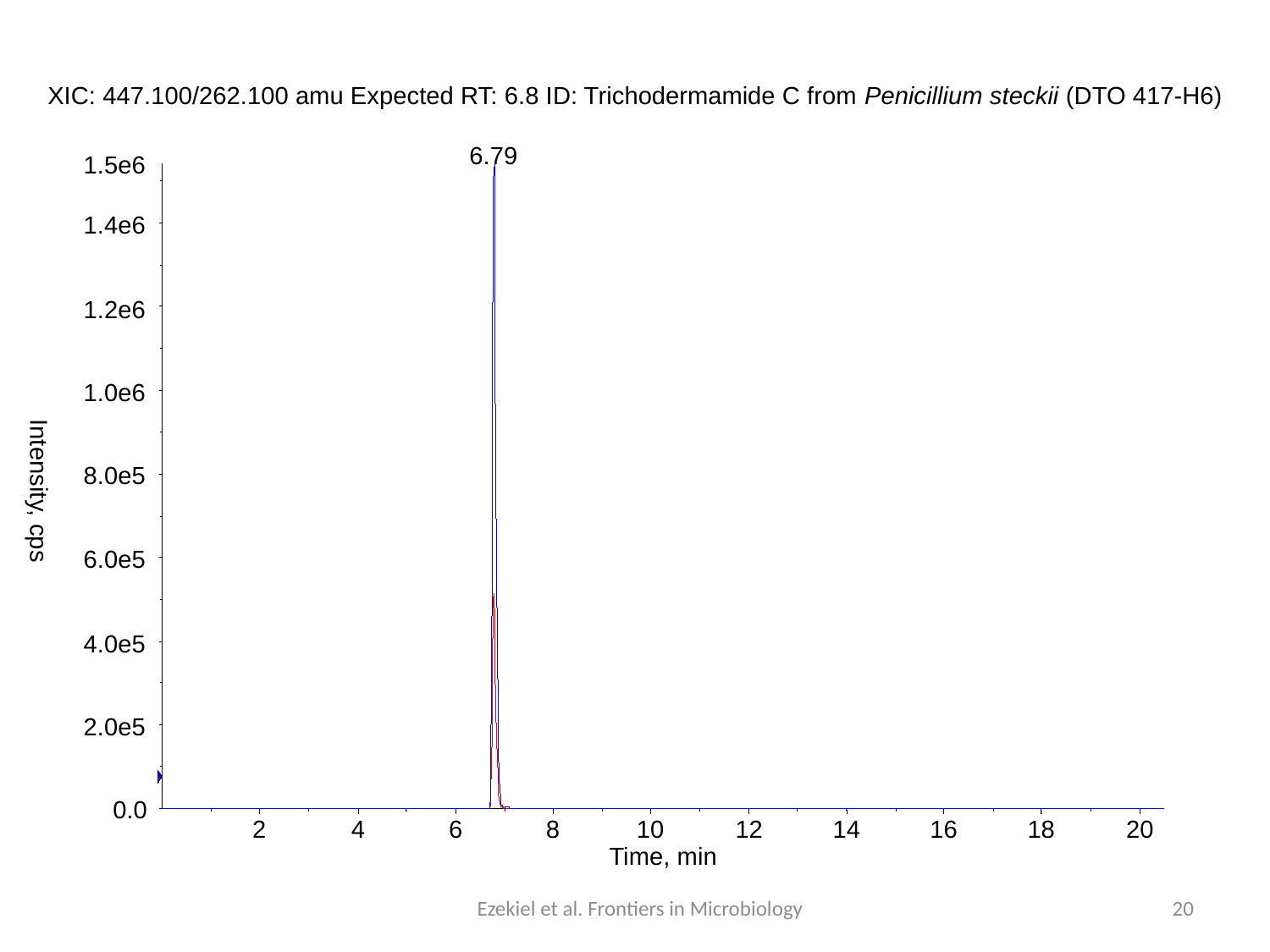

XIC: 447.100/262.100 amu Expected RT: 6.8 ID: Trichodermamide C from Penicillium steckii (DTO 417-H6)
6.79
1.5e6
1.4e6
1.2e6
1.0e6
8.0e5
Intensity, cps
6.0e5
4.0e5
2.0e5
0.0
2
4
6
8
10
12
14
16
18
20
Time, min
Ezekiel et al. Frontiers in Microbiology
20

## Slide 21
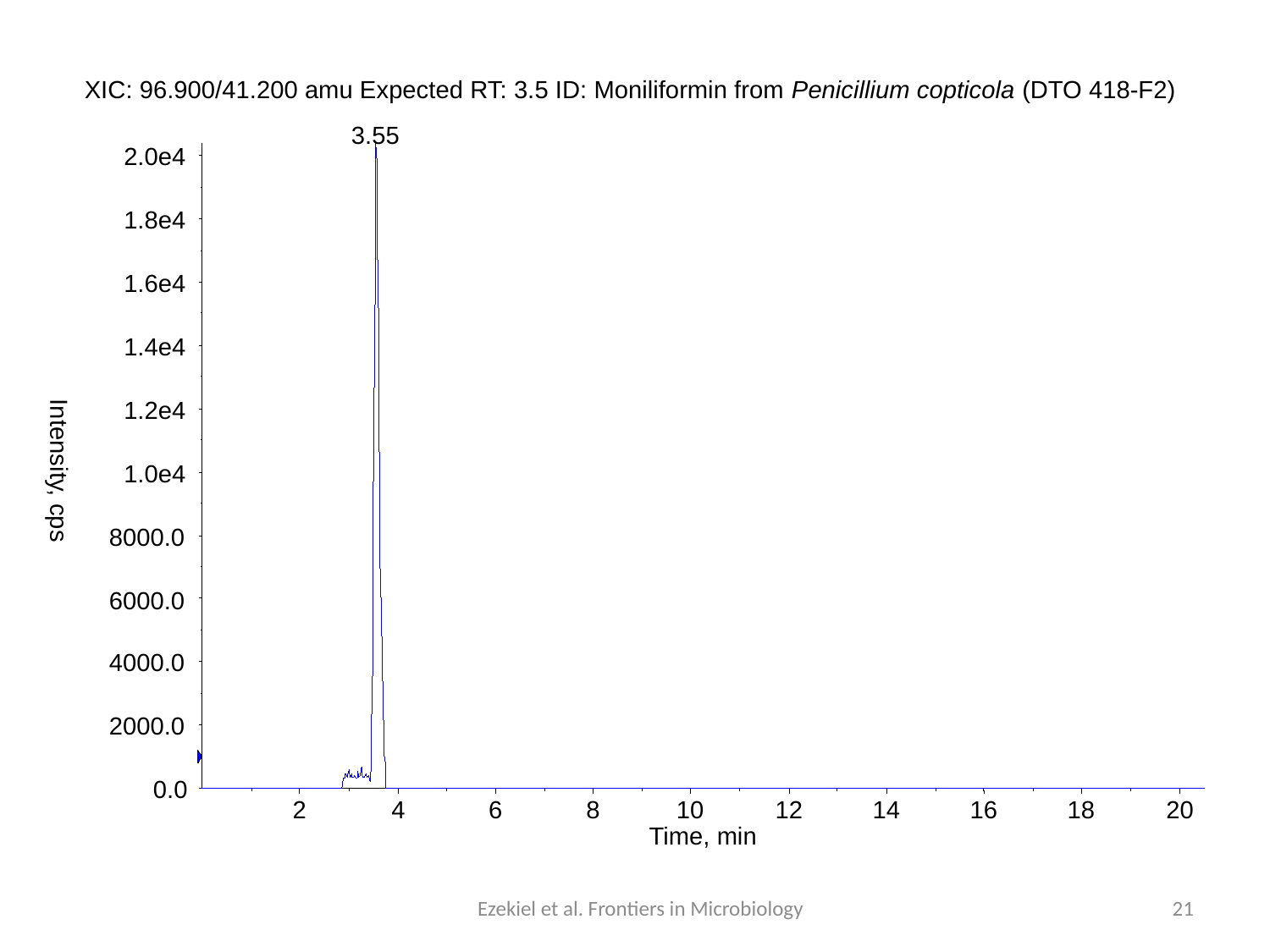

XIC: 96.900/41.200 amu Expected RT: 3.5 ID: Moniliformin from Penicillium copticola (DTO 418-F2)
3.55
2.0e4
1.8e4
1.6e4
1.4e4
1.2e4
Intensity, cps
1.0e4
8000.0
6000.0
4000.0
2000.0
0.0
2
4
6
8
10
12
14
16
18
20
Time, min
Ezekiel et al. Frontiers in Microbiology
21

## Slide 22
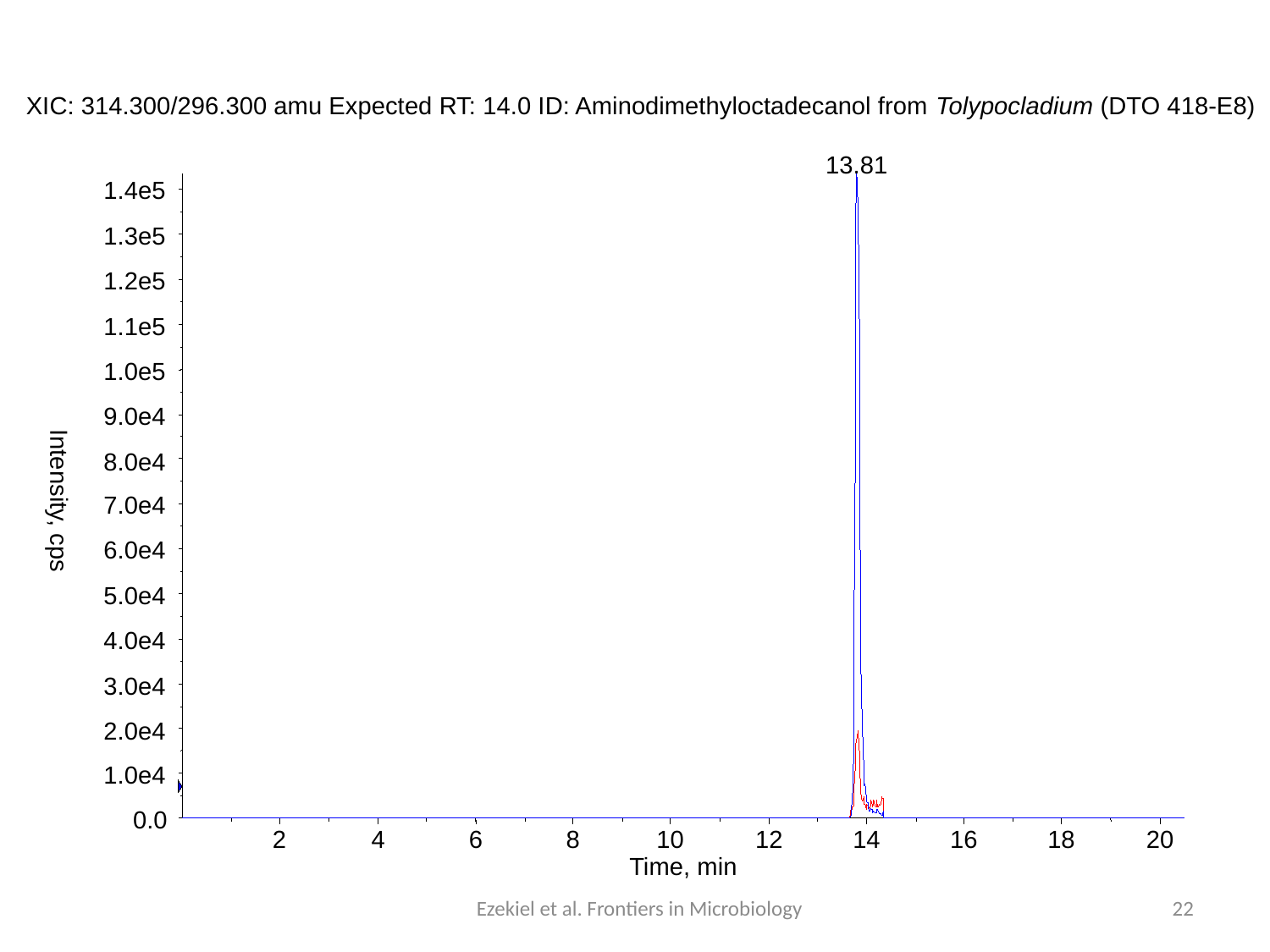

XIC: 314.300/296.300 amu Expected RT: 14.0 ID: Aminodimethyloctadecanol from Tolypocladium (DTO 418-E8)
13.81
1.4e5
1.3e5
1.2e5
1.1e5
1.0e5
9.0e4
8.0e4
Intensity, cps
7.0e4
6.0e4
5.0e4
4.0e4
3.0e4
2.0e4
1.0e4
0.0
2
4
6
8
10
12
14
16
18
20
Time, min
Ezekiel et al. Frontiers in Microbiology
22
